# Mixed lineage kinase ZAK promotes epithelial–mesenchymal transition in cancer progression

**DOI:** 10.1038/s41419-017-0161-x

**Published:** 2018-02-02

**Authors:** Linna Li, Ning Su, Ting Zhou, Dayong Zheng, Zheng Wang, Haoyu Chen, Shoujun Yuan, Wenliang Li

**Affiliations:** 1 Department of Pharmacology and Toxicology, Beijing Institute of Radiation Medicine, Beijing, 100850 China; 2 Texas Therapeutics Institute, Brown Foundation Institute of Molecular Medicine, University of Texas Health Science Center at Houston, Houston, TX 77030 USA; 3 Department of Oncology, Guangzhou Chest Hospital, Guangzhou, Guangdong China; 4 Department of Pharmacy, Fengxian Hospital, Southern Medical University, Shanghai, China; 5 Department of Medical Oncology, Nanfang Hospital, Southern Medical University, Guangzhou, China; 6 Division of Oncology, Department of Internal Medicine, University of Texas Health Science Center at Houston, Houston, TX 77030 USA; 7 The University of Texas MD Anderson Cancer Center UTHealth Graduate School of Biomedical Sciences, Houston, TX 77030 USA

## Abstract

ZAK, a mixed lineage kinase, is often described as a positive or negative regulator of cell growth. We identified it as one of the top hits in our kinome cDNA screen for potent regulators of epithelial mesenchymal transition (EMT). Ectopic expression of ZAK promoted EMT phenotypes and apoptosis resistance in multiple epithelial cell lines, while having different impacts on cell growth in different cell lines. Conversely, depletion of ZAK in aggressive mesenchymal cancer cells reversed EMT phenotypes, increased sensitivity to conventional cytotoxic drugs, and attenuated bone metastasis potential, with little impact on primary tumor growth. Mechanistically, ZAK-mediated EMT is associated with activation of ZEB1 and suppression of epithelial splicing regulatory proteins (ESRPs), which results in a switch in CD44 expression from the epithelial CD44v8–9 isoform to the mesenchymal CD44s isoform. Of note, transcriptomic analysis showed that ZAK overexpression is significantly associated with poor survival in a number of human cancer types. Tissue microarray analysis on breast invasive carcinoma further supported that ZAK overexpression is an independent poor prognostic factor for overall survival in breast cancer. Through combination with ZAK, prognostic accuracy of other common clinicopathological markers in breast cancer is improved by up to 21%. Taken together, these results suggest that promoting EMT is the primary role for ZAK in cancer progression. They also highlight its potential as a biomarker to identify high-risk patients, and suggest its promise as a therapeutic target for inhibiting metastasis and overcoming drug resistance.

## Introduction

The epithelial–mesenchymal transition (EMT), which confers mesenchymal properties on epithelial cells is an essential process in embryonic development, wound healing, organ fibrosis, and cancer progression^1,2^. In tumors of epithelial origin, aberrant induction of EMT contributes to tumor invasion and metastasis^3–5^. Increasing evidence indicates EMT also bestows tumor cells with cancer stem cell (CSC)-like characteristics, enabling therapeutic resistance and tumor recurrence^6–8^. However, our knowledge of this critical process is still quite limited, especially with respect to identification of druggable regulators. Since kinases have been established as promising drug targets, we carried out a human cDNA library screen on 500 human kinases and identified a number of potential new EMT regulators^9^. Leucine-zipper and sterile-α-motif kinase (ZAK) was one of top hits from the EMT cDNA screen. In this study, we set out to examine a critical role of ZAK in promoting EMT and cancer progression.

ZAK, also known as ZAK-α or MLK-like MAP triple kinase-α (MLTK-α), belongs to a subfamily of MAP3Ks referred to as mixed-lineage kinases (MLKs)^10–12^. ZAK was first described as a tumor suppressor gene^10,13–16^, inhibiting proliferation of human lung cancer cells^14^, inducing apoptosis of Hep3B hepatoma cells^10^, and mediating doxorubicin-induced and UV-induced apoptotic responses in pseudo-normal keratinocyte cell line HaCaT^15,16^. Recently, increasing evidence supports its pro-oncogenic functions^17–23^. Ectopic expression of ZAK effectively induces proliferation of skin epidermal cells^17^ and stimulates anchorage-independent colony growth of murine fibroblasts NIH-3T3^18^. Furthermore, ZAK-overexpressing cells forms fibrosarcomas when injected subcutaneously into immunodeficient mice^17,18^. Conversely, depletion of ZAK expression in SW620 colon cancer cells results in growth reduction of xenograft colon tumors^18^. Together, the controversial roles of ZAK on cell growth suggest that regulating cell proliferation may not be the primary role of ZAK in cancer progression. The key role of ZAK in cancer progression remains unclear.

In this study, we establish ZAK as a potent promoter for EMT. Ectopic expression of ZAK in epithelial cell lines was characterized by defined EMT features and distinctive stem-like properties. Conversely, depletion of ZAK in mesenchymal cancer cells resulted in a reversal of EMT and inhibition of bone metastasis. With regard to clinical implications, analyzes on The Cancer Genome Atlas (TCGA) database and tissue microarray (TMA) showed that ZAK overexpression is associated with poor overall survival, especially for breast invasive carcinoma patients. Collectively, these results shed new light on the key role of ZAK in cancer progression.

## Results

### ZAK induces EMT and stem cell-like properties in epithelial cell lines

**Fig. 1:**
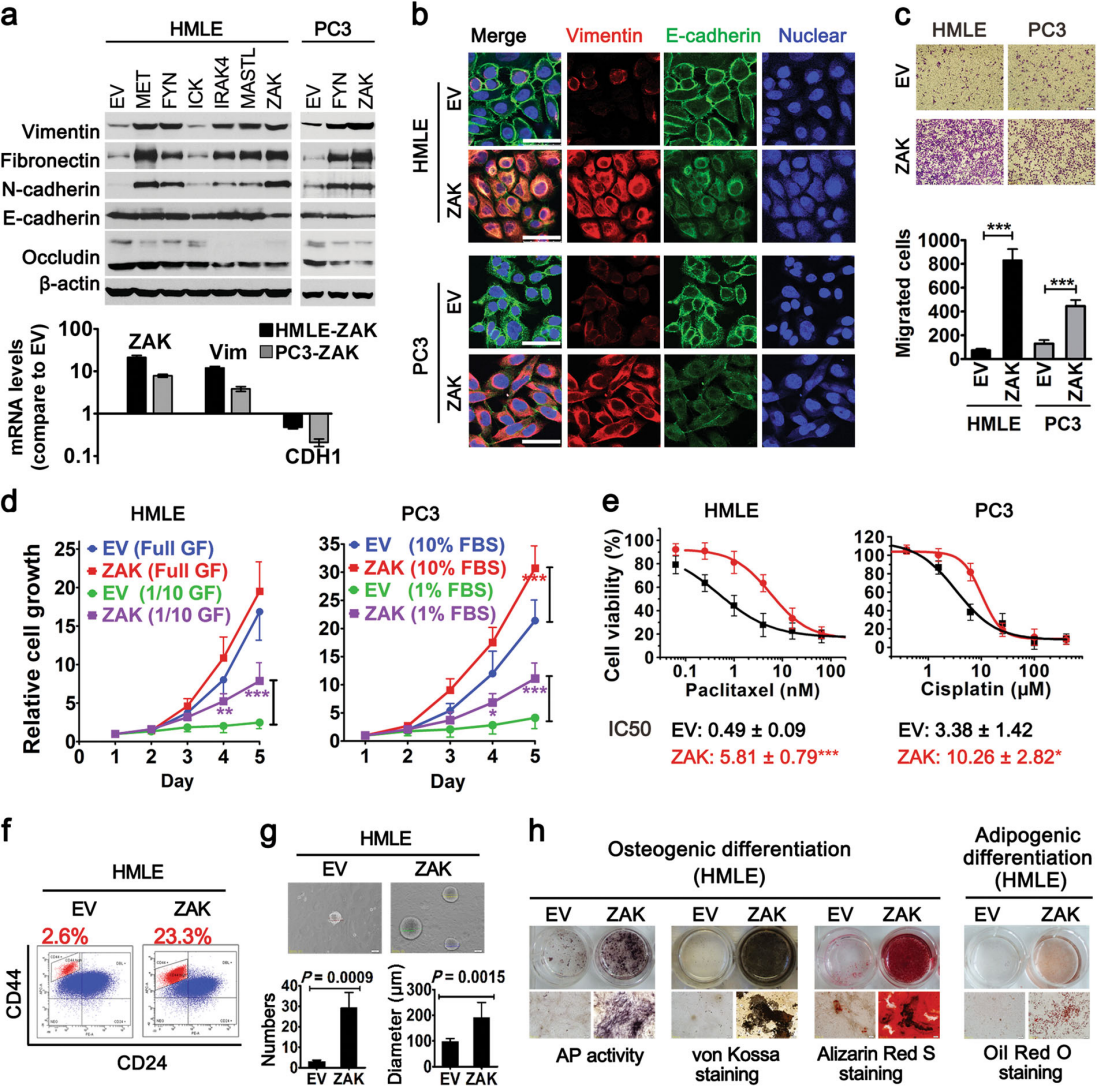
ZAK induces EMT and stem cell-like properties in epithelial cell lines **a** Changes in the expression of EMT markers upon ZAK overexpression. Top, western blot analysis of mesenchymal markers (vimentin, fibronectin, and N-cadherin) and epithelial markers (E-cadherin and occludin) in stable cell lines expressing ZAK, other candidate EMT kinases, positive kinase controls (MET and FYN), and empty vector control (EV). Bottom, RT-PCR analysis of ZAK, vimentin, and E-caderin (CDH1) expression. Shown on the *Y*-axis is log10-fold change of mRNA level induced by ZAK vs. vector control. Error bars denote SD from triplicate. **b** Immunofluorescence staining patterns of vimentin and E-cadherin in HMLE and PC3 cells expressing EV or ZAK. Cell nuclei were stained with DRAQ5. Scale bars, 50 μm. **c** ZAK promoted migration as determined by Boyden chamber assay, showing representative photos (top) and quantification (bottom) of migration. Data are represented as mean ± SD of triplicate experiments. ***P < 0.001 (Student’s *t*-test) **d** Proliferation curves of epithelial cell lines expressing ZAK or EV grown in regular media or growth factor reduced medium. GF growth factors. The number of viable cells was measured by AlamarBlue assay at different time points and data are expressed as viable cell number and fold changes from that at Day 1. Mean values ± SD from three independent experiments are shown. *P < 0.05, **P < 0.01 and ***P < 0.001 (Two-way ANOVA followed by Bonferroni post-tests). **e** Dose-response curves of indicated cell lines treated with chemotherapeutic drugs. IC50 values were obtained using logistic nonlinear regression analyzing model of MicroCal Origin 8.5 software. Error bars denote SD from two independent experiments, each done in quadruplicate. *P < 0.05 and ***P < 0.001 (Student’s *t*-test). **f** FACS analysis of CD44 and CD24 in HMLE-EV and HMLE-ZAK cells. The percentage of CD44^high^/CD24^low^ stem-like subpopulation is indicated. **g** HMLE-ZAK cells generated more and bigger mammospheres than HMLE-EV control cells, showing representative photos (top) and quantification of numbers and diameters of mammospheres from three independent experiments (bottom). Data are represented as mean ± SD of triplicate experiments. Statistical analysis was done using Student’s *t*-test. **h** HMLE cells expressing ZAK gained mesenchymal stem cell-like capabilities for multilineage differentiation. Following culture in osteoblastic differentiation media, cells were tested for alkaline phosphatase (AP) activity, or analyzed by silver nitrate (Von-Kossa) staining and alizarin red S staining to determine mineral deposition and calcium deposition. Following culture in adipogenic differentiation media, cells were stained with oil red dye to detect oil droplets formation. Additional data related to Fig. 1 are in Supplementary Figure S1

Previously, to identify novel regulators of EMT, we carried out a human cDNA library screen on 500 human kinases by vimentin promoter luciferase assay and identified 55 potential EMT inducers^9^. ZAK was one of the top hits of novel EMT activators^9^. In this study, to validate the role of ZAK in promoting EMT, EMT-associated assays were carried out. First, we confirmed that ectopic expression of ZAK effectively induced mesenchymal phenotypes in three epithelial cell lines (human mammary epithelial cell line HMLE, prostate cancer cell line PC3 and pancreatic cancer cell line SU86.86). Consistent with the screening result, ZAK and positive kinase controls (FYN^24^ and MET^25^) dramatically up-regulated the expression of mesenchymal markers (Fig. 1a and Supplementary Figure S1a). At the same time, ZAK induced down-regulation of epithelial markers, particularly in HMLE and PC3 cell lines (Fig. 1a). Of note, immunofluorescent staining showed that subcellular distribution of E-cadherin also changed upon ZAK overexpression (Fig. 1b). In vector control cells, E-cadherin was predominantly distributed at the membrane, showing tight cell-to-cell adhesion (Fig. 1b). In contrast, an obvious translocation of E-cadherin from membrane to cytoplasm was observed in ZAK-transduced cells (Fig. 1b), suggesting breakdown of epithelial barrier. Functionally, ZAK-transduced cells showed higher migratory ability than the vector control cells in a Boyden chamber assay (Fig. 1c and Supplementary Figure S1b). Taken together, these results indicate the overexpression of ZAK in epithelial cells effectively induces EMT.

Cancer cells undergoing EMT have also been shown to be resistant to many hostile factors, including growth factor deprivation and anticancer treatments^26,27^. We next investigated whether ZAK plays a role in resistance to starvation and chemotherapy. When grown in nutrition reduced media (containing 1% FBS or 1/10 of growth factors in regular medium), ZAK-transduced cells showed more robust survival and proliferative capacities than control cells (Fig. 1d and Supplementary Figure S1c). Furthermore, these ZAK-transduced cells were more resistant to chemotherapeutic drugs, showing 3–12-fold increases in IC50 values, as compared with the vector control cells (Fig. 1e and Supplementary Figure S1d). However, when grown in regular culture media, different cell lines showed different proliferative alterations upon ZAK overexpression. PC3-ZAK cells grew faster than control cells, whereas the proliferation of HMLE and SU86.86 cells was unaffected by ZAK overexpression (Fig. 1d and Supplementary Figure S1c). These results suggest regulating cell proliferation may not be the primary role of ZAK. EMT-associated resistance to starvation and chemotherapy may be a more relevant function for ZAK in cancer progression.

EMT has been associated with acquisition of stem cell-like properties, including expression of the putative breast CSC marker CD44^high^/CD24^low^ ^28^. To determine whether ZAK confers stem cell-like properties to mammary epithelial cells, we used flow cytometry to compare proportions of CD44^high^/CD24^low^ subpopulations in HMLE-EV and HMLE-ZAK cells. As shown in Fig. 1f, ZAK overexpression results in an approximately nine-fold increase in CD44^high^/CD24^low^ stem-like subpopulation, relative to empty vector control. We next examined the ability of HMLE-ZAK cells to form mammospheres, an in vitro measure of stemness^29^. Significantly, ectopic expression of ZAK enhanced the mammosphere-forming ability of HMLE cells, showing an approximate ten-fold increase in sphere numbers and an approximate 2-fold increase in sphere diameters (Fig. 1g). Furthermore, ZAK overexpression was capable of inducing HMLE cells to acquire mesenchymal stem cell (MSC) phenotypes, such as undergoing osteoblast and adipocyte differentiation in corresponding differentiation media (Fig. 1h). Taken together, these results establish an important role for ZAK in promoting EMT phenotypes and stem cell-like properties of epithelial cell lines.

### Candidate ZAK-induced EMT transcriptional factors

**Fig. 2:**
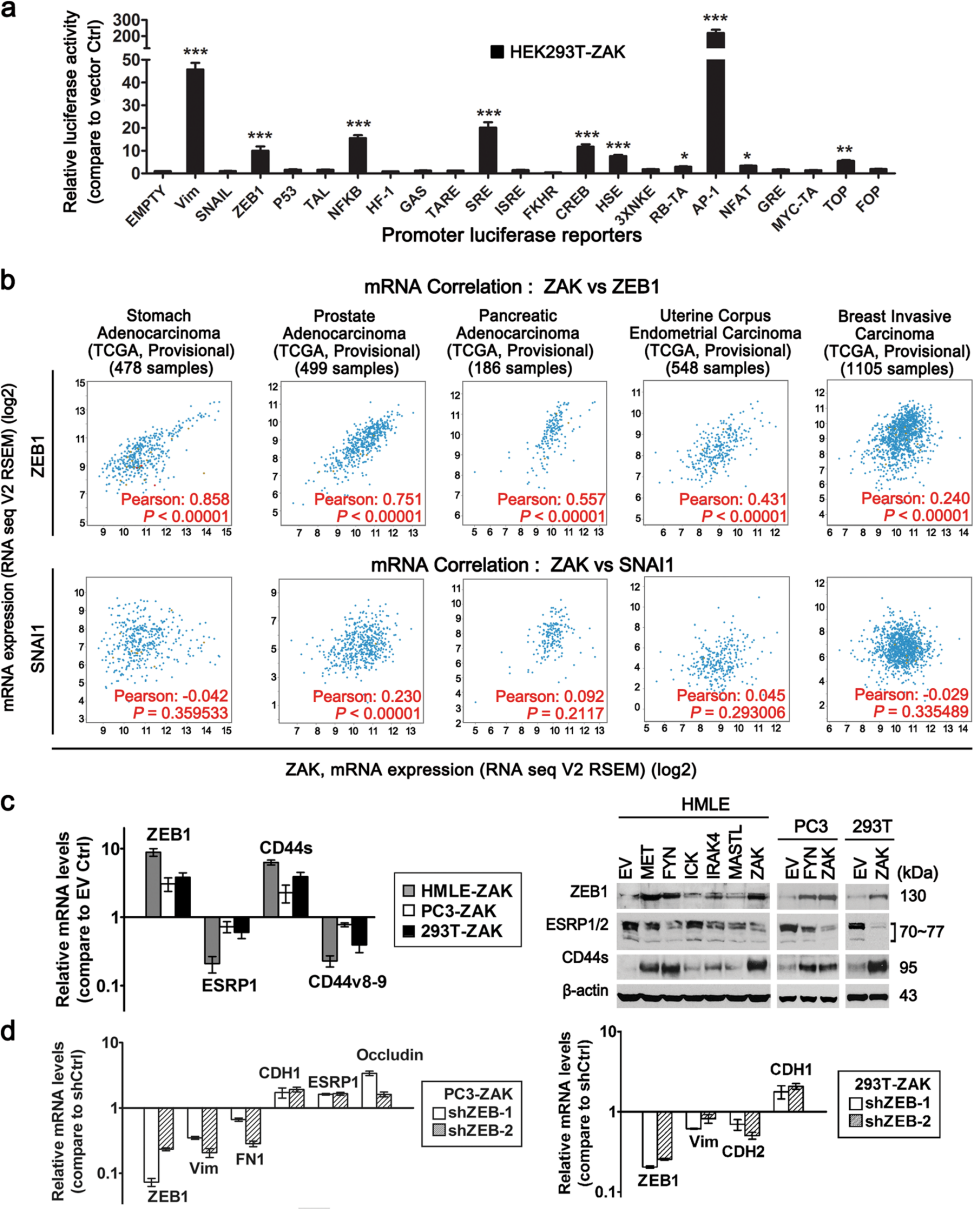
Candidate ZAK-induced EMT transcriptional factors **a** Relative luciferase activities of 23 promoter luciferase reporter constructs transiently co-transfected with ZAK or vector control, along with TK-renilla luciferase vector (pRL-TK, transfection control) into HEK293T cells. Shown on the *Y*-axis is fold changes of promoter luciferase activities induced by ZAK vs. vector control. Error bars denote SD from experiments performed in triplicate. *P < 0.05, **P < 0.01, ***P < 0.001 (One-way ANOVA test with Tukey’s post hoc test). **b** Scatter plots obtained from cBioPortal TCGA database show correlation of ZAK mRNA expression with ZEB1 and SNAI1 mRNA expression (accessed in August 2016). Pearson’s correlation coefficients *R* and *P*-values are indicated. **c** ZAK-induced alterations of ZEB1 and downstream genes in HMLE, PC3 and HEK293T cells determined by real time PCR (left) and Western Blot (right) analysis. Error bars denote SD from triplicate. **d** shRNA silencing of ZEB1 gene and resultant reversal of EMT gene expression in PC3-ZAK and 293T-ZAK cells. Changes in the expression of ZEB1 and EMT markers were determined by real time PCR. Error bars denote SD from triplicate. These experiments were repeated twice with similar results. Additional data related to Fig. 2 are in Supplementary Figure S2

Next, we sought to identify transcription factors involved in ZAK-promoted EMT. We used promoter analysis with luciferase reporters to examine the activities of a panel of transcription factors known to be involved in cancer. Among the 23 luciferase reporters, AP1 was the most activated upon ZAK overexpression, showing a more than 200-fold increase in luciferase activity (Fig. 2a). However, it is inconclusive whether AP1 is required for ZAK-induced EMT, because effective silencing two of key components of AP1 complex, c-FOS and FOSL1, in ZAK-overexpressing PC3 and 293T cells yielded non-concordant expression changes of EMT markers (Supplementary Figure S2a).

Other significantly activated reporters included ZEB1, NF-κB, cAMP responsive element (CREB), serum response element (SRE), heat shock response element (HSE), and TOPFlash (TCF4/β-catenin promoter of Wnt pathway) (Fig. 2a). Next, cBioPortal TCGA database^30,31^ was used to examine the correlation between ZAK and the above transcriptional factors in clinical cancer samples. Among the many types of human cancers examined, ZEB1 was the transcription factor that most consistently and significantly showed strong positive correlation with ZAK at the mRNA level, better than other classic EMT transcription factors, such as Snail 1 (SNAI1), Snail 2 (SNAI2, Slug), Twist 1, and Twist 2 (Fig. 2b, Supplementary Figure S2b; Supplementary Table S1). For this reason, we decided to focus on ZEB1 for its potential role as a key EMT transcription factor downstream of ZAK.

Cell culture experiments confirmed that ZEB1 was upregulated by ZAK overexpression at both mRNA and protein levels (Fig. 2c and Supplementary Figure S2c). Given that ZEB1 represses the expression of ESRPs^32^ that regulate CD44 alternative splicing during EMT^33^, we examined expression of ESRP and CD44 genes. Decreased expression of ESRP1/2 was evident in ZAK-transduced cells. Further, a switch in expression of CD44 isoforms, down-regulation of epithelial isoform CD44v8–9 and up-regulation of mesenchymal isoform CD44s, was observed in these cells (Fig. 2c). These results support the role of ZAK in ZEB1 activation and shed the light on the mechanism of ZAK-induced increase in the CD44^high^/CD24^low^ stem-like subpopulation during EMT (Fig. 1f). When the increased ZEB1 expression in PC3-ZAK and 293T-ZAK cells was inhibited by effective ZEB1 shRNAs, reversed EMT phenotypes were observed (Fig. 2d and Supplementary Figure S2d), which indicated a critical role for ZEB1 in mediating ZAK-induced EMT.

Since ZAK is one of the MAP3K family members, we next determined whether MAPK pathway is critical for ZAK-induced EMT marker expression. Treating PC3-ZAK and 293T-ZAK cells with MAPK pathway inhibitors, either U0126 or RO5126766, led to upregulation of epithelial regulators, such as ESRP1/2, and downregulation of mesenchymal genes, such as CDH2 and ZEB1 (Supplementary Figure S2e). This result suggests that MAPK pathway may be important for ZAK-induced EMT phenotypes. To investigate to what extent the kinase activity of ZAK is required for its role in promoting EMT, we transduced a ZAK kinase dead cDNA (with a K45M mutation^23^) into PC3 and 293T cells, using GFP and ZAK wild type cDNA as controls. As compared to GFP control, ZAK wild type cDNA can, while ZAK-K45M cDNA cannot, induce EMT gene expression changes (Supplementary Figure S2f). This result suggests that ZAK’s kinase activity may be crucial for its role in EMT.

### Silencing of ZAK gene in mesenchymal cells reverses EMT phenotypes and attenuates bone metastasis formation

**Fig. 3:**
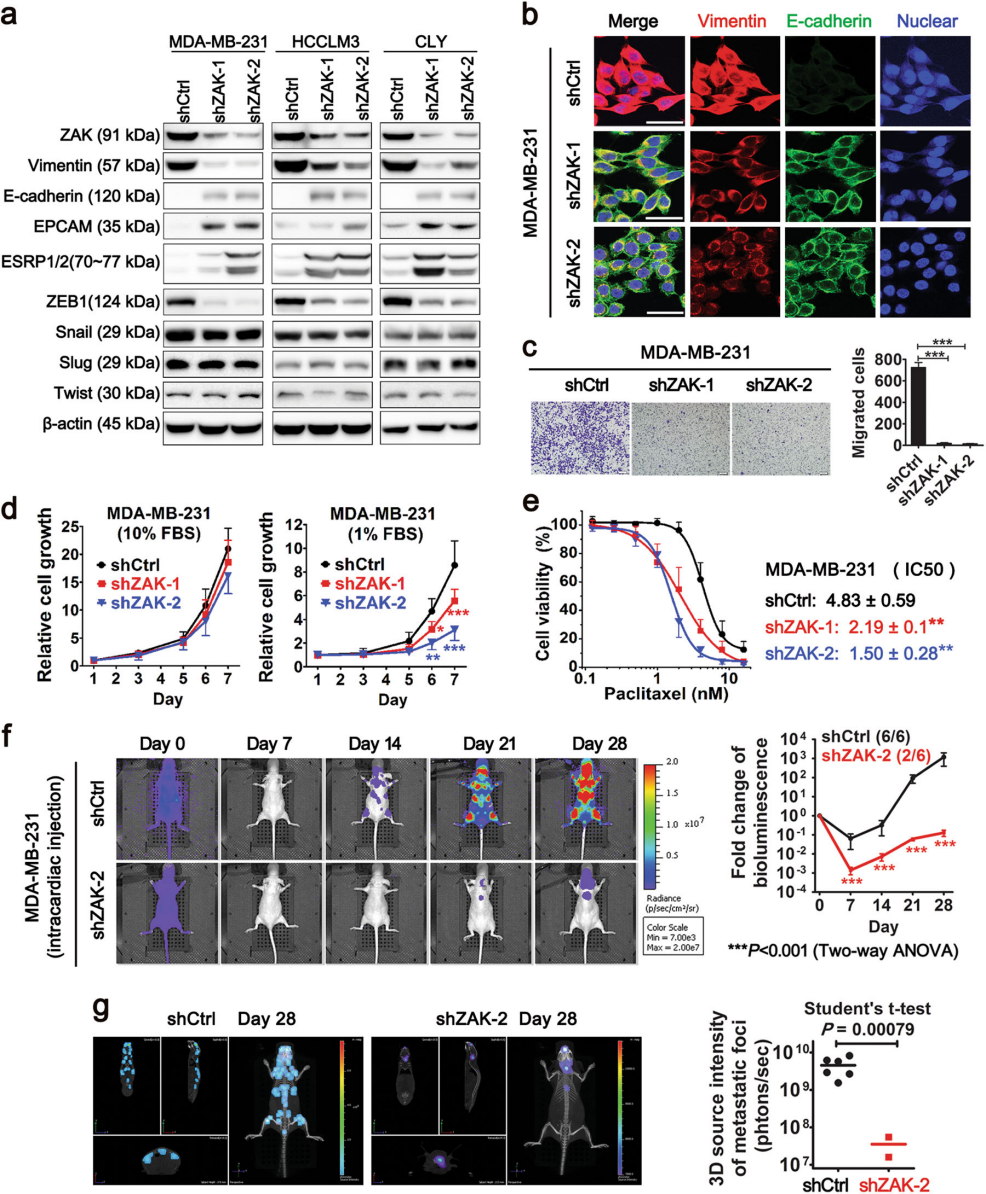
Silencing of ZAK gene in mesenchymal cells reverses EMT phenotypes and attenuates bone metastasis **a** Changes in the expression of ZAK and EMT-associated genes by ZAK knockdown in mesenchymal cells as determined by Western Blots. **b** Immunofluorescence staining patterns of vimentin and E-cadherin in MDA-MB-231 cells expressing shCrtl or shZAKs. Cell nuclei were stained with DRAQ5. Scale bars = 50 μm. **c** Representative migration photos of the above cells determined by Boyden Chamber assay. Quantification of migration is shown as means (SD from three independent experiments). ***P < 0.001 (One-way ANOVA followed by Tukey’s test). **d** Proliferation curves of the above cells grown in regular (left) or nutrition reduced media (right). Mean values ± SD from three independent experiments are shown. *P < 0.05, **P < 0.01 and ***P < 0.001 (Two-way ANOVA followed by Bonferroni post-tests). **e** Dose-response curves of the above cells treated with graded concentrations of paclitaxel. IC50 values were obtained by using logistic nonlinear regression analyzing model of MicroCal Origin 8.5 software. Error bars denote SD from two independent experiments, each done in quadruplicate. **P < 0.01 (One-way ANOVA followed by Tukey’s *post hoc* test). **f** MDA-MB-231-shCtrl cells and MDA-MB-231-shZAK2 cells were injected into left cardiac ventricle of immunedeficient mice (*n* = 6), respectively. Representative 2D bioluminescence imaging photos of the two groups at different time points are shown in the left panel. Right graph represents fold changes of bioluminescence values at each time point, which were calculated using Day 0 values as baseline and are plotted as Means ± SD **g** Representative 3D bioluminescence plus micro CT images of the above shCtrl mice and shZAK-2 mice on Day 28. Quantification of 3D bioluminescent sources is shown on right panel. Additional data related to Fig. 3 are in Supplementary Figures S3 and S4

Next, we sought to functionally validate the role of ZAK in cancer cells with high metastatic potential. To this end, breast cancer cell line MDA-MB-231, hepatocellular cancer cell line HCCLM3^34^, and colorectal cancer cell line CLY^35^, each with high levels of ZAK, ZEB1 and mesenchymal markers (Supplementary Figure S3a), were used to establish stable cell lines expressing shRNAs targeting ZAK. Upon ZAK depletion, a downregulation of vimentin and an upregulation of E-cadherin (CDH1) were observed, as determined at both mRNA and protein levels (Fig. 3a and Supplementary Figure S3b). These changes of EMT markers were also verified by immunofluorescent confocal analysis (Fig. 3b). Furthermore, effective ZAK shRNAs induced a downregulation of ZEB1 (Fig. 3a and Supplementary Figure S3b), which was consistent with the induction of ZEB1 by ZAK ectopic expression (Fig. 2c). Consistent with our observations in TCGA human tumor samples suggesting a particularly close relationship between ZAK and ZEB1, we also found that several other well-characterized EMT transcription factors, such as Snail 1, Snail 2, and Twist 1, are largely unchanged upon silencing ZAK in these mesenchymal cancer cells (Fig. 3a).

Concomitant with ZEB1 downregulation, an increased expression of ESRP1/2 and a switch in CD44 alternative splicing (from mesenchymal CD44s isoform to epithelial CD44v8–9 isoform) were evidenced in cells depleted of ZAK (Fig. 3a and Supplementary Figure S3b). Functionally, loss of ZAK markedly attenuated the migration ability of metastatic cancer cells (Fig. 3c and Supplementary Figure S3c). This reduction of migration was not due to reduced cell proliferation, as effective ZAK shRNAs did not change the proliferation rate of cancer cells grown (Fig. 3d and Supplementary Figure S3d). Nevertheless, when cultured in nutrition-reduced media (containing 1% FBS), cancer cells with ZAK depletion showed significantly weaker proliferation capacity (Fig. 3d and Supplementary Figure S3d). Moreover, resistance of cancer cells to paclitaxel was also significantly reduced by ZAK silencing (Fig. 3e and Supplementary Figure S3e). Taken together, these results indicate that ZAK depletion results in a reversal of EMT and reduced drug resistance in aggressive cancer cells.

To determine whether ZAK silencing inhibits in vivo metastatic ability of MDA-MB-231 cells, bioluminescence imaging (BLI) was used to monitor and compare metastatic tumor growth caused by MDA-MB-231-shCtrl cells and MDA-MB-231-shZAK cells. Both cells lines were transduced to express firefly luciferase (luc2), and then injected into the left cardiac ventricle of immunodeficient nude mice (*n* = 6). Ten minutes after injection (Day 0), diffuse whole-body bioluminescent signals were detected, indicating the success of left ventricular injection (Fig. 3f). Most signals disappeared at day 7 in both shZAK and shCtrl groups (Fig. 3f). However, at day 14, all shCtrl mice developed obvious bioluminescent signals that overlapped with bony structures (6/6) (Fig. 3f). In contrast, no visible signal in shZAK mice could be identified until day 21, and then, only in two mice (2/6) (Fig. 3f). At the end of the animal experiment (Day 28), all shCtrl mice (6/6) developed bone metastases. In three dimensional BLI and micro CT imaging, these mice showed clear bioluminescent foci in bony structures, including distal femurs, proximal tibiae, bony pelvis, scapula, vertebrae, distal ulnas, ribs, and skull (Fig. 3g). Two of shZAK mice (2/6) developed metastasis in the skull and vertebrae. The 3D source intensity (photon/s) of these mice was lower by two orders of magnitude than that of the shCtrl mice (Fig. 3g). However, there was no significant difference in subcutaneous tumor formation between control and ZAK knockdown MDA-MB-231 cells (Supplementary Figure S4). These results suggest that ZAK-mediated migration, invasion and survival in hostile foreign environment, rather than general effects on cell growth, contribute to the inhibitory effects of ZAK silencing on experimental metastasis of MDA-MB-231 cells.

### ZAK overexpression is significantly associated with poor prognosis

**Fig. 4:**
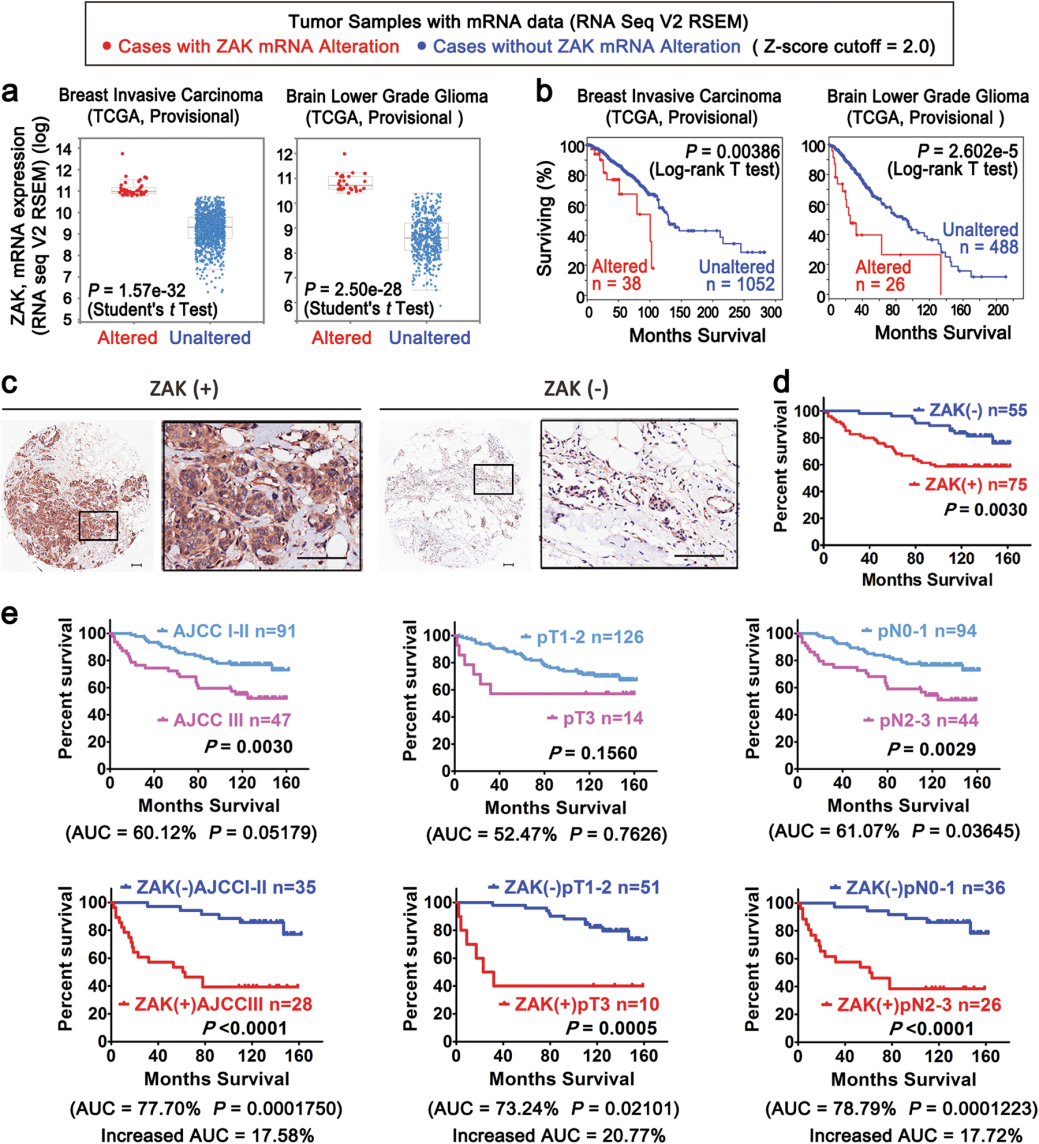
ZAK overexpression is significantly associated with poor prognosis **a** Comparison of ZAK mRNA expression between altered group and unaltered group in two TCGA datasets. **b** Kaplan-Meier survival curves depending on ZAK mRNA alterations in above TCGA datasets. All plots and statistical analyses in (**a**, **b**) were obtained from cBioPortal TCGA database (www.cbioportal.org) in August, 2016. More datasets are shown in Supplementary Figures S6. **c** Breast invasive carcinoma samples in the TMA were immunostained with an anti-ZAK antibody. Representative ZAK-positive and ZAK-negative samples are shown. Scale bars = 50 μm. **d** Kaplan–Meier curves for overall survival differences between ZAK-positive and ZAK-negative groups. **e** Combination of ZAK with clinicopathological parameters results in more accurate stratification and prognosis of breast invasive cancer cases in the TMA dataset. Kaplan–Meier overall survival curves on individual clinicopathological parameters (top), and in combinations with ZAK (bottom). The *P*-value was calculated using the Log-rank test. AUCs obtained from ROC analysis represented the prognostic accuracy. Additional data related to Fig. 4 are in Supplementary Figures S5, S6, and S7

In vitro and in vivo results described above suggested that ZAK promotes cancer progression. To assess clinical implications of these results, we examined ZAK expression and its correlation with patient prognosis in TCGA profiling data for human solid cancer types. A cross-cancer alteration summary was derived from the cBioPortal website^30,31^ (Accessed in August, 2016). This summary showed that amplification at the DNA level and upregulation at the mRNA level were the major alterations of ZAK in a number of human solid cancer types, accounting for 16 and 61% respectively, in ZAK-altered cases (Supplementary Figure S5). Furthermore, ZAK mRNA upregulation was associated with significantly reduced survival in many TCGA solid cancer types, such as breast invasive carcinoma, brain lower grade glioma, cervical squamous cell carcinoma, lung adenocarcinoma, and renal papillary cell carcinoma (Figs. 4a, b and Supplementary Figure S6).

Next, we examined the expression of ZAK protein in 140 breast invasive carcinoma samples in a TMA (clinicopathological information is shown in Supplementary Table S3), to determine the extent to which ZAK can be an important factor in determining clinical outcomes of breast invasive carcinoma. Results of immunohistochemistry (IHC) assays revealed that ZAK is primarily located in the cytoplasm (Fig. 4c). Binary ZAK staining was designated positive (+) or negative (−) staining, as shown in Fig. 4c. Kaplan–Meier analyses showed that positive ZAK protein expression was associated with significantly reduced overall survival (Fig. 4d), consistent with TCGA transcriptomic data (Fig. 4b). We also examined other clinicopathological parameters. We observed that ZAK expression was positively associated HER2 expression (*P* = 0.0214, relative risk = 1.793), and negatively associated with expression of estrogen receptors (ER) (*P* = 0.0002, relative risk = 0.3556) and progesterone receptors (PR) (*P* < 0.0001, relative risk = 0.2596) (Supplementary Table S4). Among different intrinsic subtypes of breast cancers, HER2 type and basal-like breast cancer cases showing negative ER and PR expression were strongly correlated with positive ZAK expression (*P* < 0.0001, relative risk = 0.2488, Supplementary Table S4).

Further univariate survival analyses demonstrated that ZAK, ER, PR, and advanced lymph node metastasis were statistically significant predictors of overall survival (Table 1 and Supplementary Table S5). In multivariate analysis, after adjusting for tumor grade, tumor stage, and other biomarker expression, positive ZAK expression (Hazard ratio = 2.775, *P = *0.006) and advanced lymph node metastasis (Hazard ratio = 2.458, *P = *0.006) were found to be independent negative prognostic factors for overall survival (Table 1 and Supplementary Table S5). Notably, combining ZAK with ER, PR, HER2, and lymph node status resulted in better stratification of patients and more pronounced effects on overall survival (Table 1, Supplementary Table S5, Fig. 4e and Supplementary Figure S7). The combination of ZAK expression with lymph node status resulted in best stratification of patients, showing a hazard ratio of 5.831 (*P* < 0.001, Table 1).

**Table 1:** COX univariate and multivariate analysis for the effect of ZAK expression on overall survival

Clinicopathological parameters	Univariate analyses		Multivariate analyses	
	Hazard ratio (95% CI)	*P*-value	Hazard ratio (95% CI)	*P*-value
*Biomarkers*				
ZAK (+)	2.726 (1.368–5.435)	0.004	2.775 (1.344–5.730)	0.006
ER (+)	0.455 (0.244–0.846)	0.013	0.843 (0.313–2.275)	0.737
PR (+)	0.507 (0.272–0.946)	0.033	0.647 (0.317–1.324)	0.233
HER2 (+)	1.023 (0.529–1.977)	0.947	0.648 (0.322–1.305)	0.224
*Lymph node status*				
pN2–3 (vs. pN0–1)	2.391 (1.323–4.322)	0.004	2.458 (1.298–4.656)	0.006
*Combination 1*				
ZAK + ER− (vs. ZAK-ER + )	3.779 (1.614–8.849)	0.002	3.417 (1.458–8.010)	0.005
*Combination 2*				
ZAK + PR− (vs. ZAK-PR + )	3.288 (1.426–7.585)	0.005	3.654 (1.514–8.821)	0.004
*Combination 3*				
ZAK + HER2− (vs. ZAK-HER2−)	3.401 (1.485–7.786)	0.004	3.637 (1.518–8.717)	0.004
*Combination 4*				
ZAK + Basal like (vs. ZAK-Luminal type)	4.509 (1.825–11.144)	0.001	4.357 (1.759–10.791)	0.001
*Combination 5*				
ZAK + pN2–3 (vs. ZAK-pN0–1)	6.538 (2.548–16.774)	<0.001	5.831 (2.234–15.224)	<0.001

Luminal type: ER+ and/or PR+; Basal like: ER-PR-HER2−; 95% CI: 95% confidence intervals

To compare prognostic accuracy of these markers, the area under the curve (AUC) of receiver operating characteristic curve (ROC) was calculated. The AUC measures are among the most commonly used indicators for discriminative ability of a prediction model^36^. The expected AUC of a random classifier is 0.5 (or 50%). An AUC of 0.5 (or 50%) is therefore commonly interpreted as an indicator for a useless model, whereas a value of 1.0 (or 100%) is associated with a perfect classifier. Among all individual clinicopathological parameters, ZAK was the most accurate prognostic indicator for overall survival (AUC = 69.67%, Table 2). Its predictive power was even comparable to that of combined ER, PR, and HER2 (AUC = 68.63%, Table 2). With the addition of ZAK, prognostic accuracy of other clinicopathological markers was improved by up to 20.77% (Table 2). Taken together, results indicate positive ZAK expression is an independent negative prognostic factor in breast invasive carcinoma. Combination of ZAK with other clinical parameters significantly improves the prognostic capability for identifying high-risk patients.

**Table 2:** ROC analysis for overall survival

Variables	AUC (%)^a^	SE (95% CI)	*P-*value^b^	Increased prognostic values (%)^c^
*Individual parameters*				
Primary tumor stage (pT1–2 vs. pT3)	52.47	11.1(30.68–74.25)	0.7626	
AJCC classification (I-II vs. III)	60.12	5.42 (49.51–70.74)	0.05179	
LN (pN0–1 vs. pN2–3)	61.07	5.43 (50.42–71.72)	0.03645	
HER2	61.38	5.08 (51.42–71.34)	0.03425	
ER	64.55	5.35 (54.06–75.04)	0.006993	
PR	66.41	4.77 (57.05–75.76)	0.001573	
ZAK	69.67	4.54 (60.78–78.57)	0.001328	
*Combined markers*				
ER, PR,and HER2 (Basal type vs. other types)	68.63	6.30 (56.28–80.98)	0.007054	
ZAK and HER2	69.76	6.05 (59.70–81.62)	0.002082	8.38
ZAK and pT	73.24	11.80 (50.09–96.38)	0.02101	20.77
ZAK and PR	73.41	5.37 (62.89–83.94)	0.0001728	7.00
ZAK and ER	75.45	5.79 (64.11–86.80)	0.0001334	10.90
ZAK and AJCC classification	77.70	6.38 (65.20–90.21)	0.0001750	17.58
ZAK and pN	78.79	6.43 (66.18–91.41)	0.0001223	17.72
ZAK, ER, PR, and HER2	80.18	6.52(67.39–92.97)	0.0001487	11.55

*ROC* receiver operating curve, *AUC* area under the curve, *SE* standard error, *95% CI* 95% confidence intervals

^a^The prognostic ability was evaluated by AUC. The AUC values range from 50 to 100%. The larger the AUC values, the more accurate the markers

^b^ROC curve analysis between AUC of indicated parameter and AUC = 50% (no prognostic value)

^c^Increased prognostic values = AUC of combined markers—AUC of corresponding individual markers (without combination of ZAK)

## Discussion

For a long time, ZAK studies have focused on its role in cell growth regulation. However, previous reports^10,13–23^ on ZAK’s negative and positive regulatory effects suggest that its cell growth regulation is context dependent and may not be its primary role in cancer progression. In this study, we demonstrated a potent role of ZAK in promoting EMT and resistance to apoptosis-inducing conditions. This result is in line with ZAK-induced cell migration observed by other investigators^22,23^. Of note, we found that depletion of ZAK in breast cancer cells MDA-MB-231 reversed their mesenchymal phenotypes and attenuated their bone metastasis potentials in mouse. Our in vivo result is consistent with an in vitro result reported by Nyati et al. They showed that DHP-2, a ZAK specific inhibitor, inhibited TGF-β-mediated cell migration in breast cancer cell line MDA-231–1833^37^. Collectively, these results suggest that ZAK may be a good target for metastasis countermeasures.

We also investigated the effect of ZAK on cell proliferation. Due to inconsistency of ZAK effects on the growth of different cell lines under the normal culture conditions, no definite conclusion could be drawn. We found that the effect of ZAK on cell growth was environment-dependent. When grown in nutrition-reduced media, ZAK-overexpressing cells exhibited enhanced starvation endurance capacity, whereas ZAK knockdown cells showed weakened survival ability. Similar results were observed in animal experiments. There was no significant difference in subcutaneous tumor formation between control and ZAK knockdown MDA-MB-231 cells. However, shZAK cells had a much lower capability to form bone metastasis. We speculate that ZAK bestows tumor cells with survival advantage in a hostile environment through its promoting EMT and resistance to apoptosis. During cancer progression, there are growing challenges in the microenvironment, characterized by hypoxia, low pH, and nutrient deprivation. Through EMT, tumor cells breakthrough the limitations of local survival, metastasize to other organs, and thrive in foreign microenvironments^38^. By ZAK activation, differentiated epithelial cancer cells can re-enter the undifferentiated mesenchymal state, acquiring stem-like features, including survival advantage in hostile conditions and foreign microenvironments.

ZAK also protects tumor cells from other hostile factors. Our results showed that ZAK overexpression results in chemotherapy resistance and ZAK depletion increases the sensitivity of aggressive cancer cells to conventional cytotoxic drugs. Similarly, Markowitz et al. demonstrated that ZAK protects tumor cells from ionizing radiation-induced cell death^39^. Conversely, both siRNA-mediated ZAK silencing and specific ZAK inhibitor M443 effectively radiosensitize cancer cells^40^. Moreover, ZAK inhibitor M443 synergizes with radiation to prolong survival in a murine model of medulloblastoma^40^. Taken together, these results suggest that ZAK inhibitors may be combined with chemotherapy or radiotherapy to lower the dose of cytotoxic drugs or radiation while maintaining therapeutic efficacy. As one of our next steps, we will acquire existing and generate new ZAK inhibitors to pursue this opportunity.

We made an initial investigation into how ZAK activates EMT and accompanying stem cell-like features. We observed that AP1, NF-κB, and β-catenin were significantly activated upon ZAK overexpression, which was consistent with previous publications^10,14,20,23^. Our results provided additional clues for the mechanisms underlying ZAK-induced EMT, including transcriptional activation of ZEB1, SRE, CREB, and HSE. We further demonstrated that ZEB1 is a key mediator of ZAK-induced EMT and kinase activity of ZAK may be crucial for its role in EMT. With the close relationship between ZAK and ZEB1, ZAK may serve as an alternative target of ZEB1 that is thought non-druggable or difficult to target for cancer therapy. However, to understand the relationship between ZAK and ZEB1 fully and exactly, further work is warranted to determine direct substrates of ZAK kinase in EMT regulation and the precise mechanism of ZEB1 activation upon ZAK overexpression.

Analyses of public transcriptomic databases and TMA support the conclusion that ZAK promotes human cancer progression. According to cBioPortal database, mRNA upregulation is the most dominant alteration of ZAK in most human cancers. Others have also reported that ZAK transcripts and proteins are frequently upregulated in gastric, breast, bladder, and colorectal cancers, relative to corresponding normal samples^19,23^. Notably, we found that ZAK overexpression is significantly associated with poor survival, especially for breast invasive carcinoma. Specifically, our TMA analysis of breast invasive carcinoma reveals the prognostic potential of ZAK as a biomarker to identify high-risk patients. First, ZAK overexpression is associated with significantly reduced overall survival. Second, ZAK is a more accurate prognostic indicator for overall survival than the ER, PR, and HER2. Third, combination of ZAK with other clinical parameters resulted in significantly better stratification of patients and even more accurate prognosis for overall survival. The current study has limited TMA sample size (*n* = 140). Validation studies with a larger set of breast cancer specimens and other cancer types are necessary to support the prognostic value of ZAK.

In summary, our results demonstrate the key role for ZAK in promoting EMT and cancer progression and highlight its potential as a biomarker to identify high-risk patients with breast invasive carcinoma. Together, the results support ZAK as a therapeutic target for inhibiting metastasis and overcoming drug resistance.

## Materials and methods

### Cell culture

The cell lines and cells culture are shown in Supplementary Information.

### Vector constructs, retro-viral and lenti-viral preparation

Construction of the retroviral vector pJP1563-ZAK containing full length of the ZAK gene has been previously described^41^. Generation of ZAK-kinase dead mutant pJP1563-ZAK-K45M was done through QuikChange site-directed mutagenesis kit (Agilent, Santa Clara, CA). Lentiviral shRNAs constructs targeting ZAK and ZEB1 were purchased from Sigma-Aldrich (St. Louis, MO, USA): pLKO.1-shZAK-1 (TRCN0000003265), pLKO.1-shZAK-2 (TRCN0000003266), pLKO.1-shZEB1-1 (TRCN0000017565), and pLKO.1-shZEB1–2 (TRCN0000017567). Vectors were packaged into viral particles in 293T cells for transduction according to previous description^41,42^.

### Western blotting and immunofluorescence assays

The details of western blotting and Immunofluorescence assays are shown in Supplementary Information.

### Reverse transcription and quantitative PCR analysis

Experiments were carried out according to the protocol practically used in our laboratory^9^. The sequence of PCR primers are shown in Supplementary Information.

### Migration assay

Cells were starved by culturing in medium without FBS overnight. After starvation, cells were split and 5 × 10^4^ cells were seeded onto transwell membrane inserts (24-well transwell plate containing 8 µm pore-size polycarbonate filters, Corning Costar, Boston, MA, USA) in FBS-free medium. Medium containing 2% FBS was added to the lower chamber. After 24 h incubation at 37 °C, non-migrating cells were wiped from the upper side of the membrane. Cells that migrated to the lower surface of the membrane were fixed and stained with 0.5% crystal violet solution (in 95% ethanol). Duplicate inserts were used for each individual experiment, and five random microscopic fields were counted per insert.

### FACS, mammoshpere, and multilineage differentiation assays

Mammoshpere and multilineage differentiation assays have been shown in Supplementary Information.

### Cell growth and cell viability assay

Cells (1000/well) were plated in 96-well plate. After 24 h, cells were gently washed with PBS and cultured with medium supplemented with decreasing concentrations of growth factors or FBS for 5–6 days. Medium with full amounts of growth factors was regarded as 100% growth factors. Cell proliferation was then measured using the AlamarBlue assay. Briefly, AlamarBlue reagent (Invitrogen) was added to each well, and the plate was incubated at 37 °C for 4 h. The plates were then read on the Tecan Infinite M1000 plate reader (TECAN Groups Ltd., Mannedorf, Switzerland) using an excitation wavelength of 535 nm and an emission wavelength of 595 nm. For drug resistance assay, all compounds were purchased from Sigma-Aldrich and dissolved in DMSO. Cells (3000/well) were plated in 100 µl per well in 96-well plates. One day (24 h) after seeding, compounds were added in quadruplicate per concentration for each cell line. Cell viability was measured after 72 h using the AlamarBlue assay.

### Promoter reporter luciferase assay

Experiment for promoter reporter luciferase assay was performed as described previously^9^ and the details are shown in Supplementary Information.

### Animal experiments

Six-week-old female athymic nude mice (Balb/c nu/nu) were purchased from Vital River (Beijing, China). All animal care and experimental procedures conformed to the Guide for the Care and Use of Laboratory Animals as adopted and promulgated by Beijing Medical Experimental Animal Care Commission. The present study was approved by The Laboratory Animal Ethics Committee of Beijing Institute of Radiation Medicine.

MDA-MB-231-shCtrl and MDA-MB-231-shZAK2 cells were transduced to express luciferase and in vitro luciferase assay was performed to ensure similar luciferase expression in both cell lines. For intracardiac injection, mice were anesthetized with 100 mg/kg ketamine and 10 mg/kg xylazine (Sigma-Aldrich), and 1 × 10^6^ cells suspended in 0.1 ml of PBS were injected into the left cardiac ventricle of nude mice using 29G needles as previously described^43^. For subcutaneous injection, 1 × 10^5^ cells suspended in 0.1 ml of PBS were injected into the back of nude mice using 26G needles. For bioluminescence imagining, mice were anaesthetized using 2% isoflurane and injected intraperitoneally with 150 mg/kg d-luciferin, and then imaged using IVIS Spectrum CT platform (PerkinElmer, Norwalk, CT, USA). Living image 4.3.1 software was used for data analysis (PerkinElmer).

### TCGA data analysis

Cross-cancer alterations for ZAK were examined in TCGA database through the cBioportal analysis platform (www.cbioportal.org). The downloaded data files (Accessed in August, 2016) included genetic alteration, mRAN expression (RNA-Seq Expectation-Maximization-normalized gene expression values), mRAN correlation between ZAK and other genes, overall survival and disease-free survival Kaplan–Meier curves, as well as corresponding statistical analyses.

### TMA and IHC

ZAK level in breast invasive carcinoma was evaluated by IHC using anti-ZAK antibody (HPA017205, Sigma) on commercial tissue array HBre-Duc140Sur-01 (Shanghai Outdo Biotech, Shanghai, China). The TMA contained 140 breast tumor specimens (all were breast invasive ductal carcinomas ranging from Stage I to Stage III). The sections were dewaxed, hydrated, and washed. After neutralization of endogenous peroxidase and microwave antigen retrieval, slides were pre-incubated with blocking serum and then were incubated with ZAK antibody (1:100) at 4 °C overnight. Subsequently, the sections were serially rinsed, incubated with second antibody (horseradish peroxidase-conjugated goat anti-rabbit IgG) for 30 min at room temperature, and treated with HRP-conjugated streptavidin. After washing, the bound antibody was visualized with 3, 3-diaminobenzidine tetrahydrochloride and the nuclei were restained with hematoxylin. The stained TMA section was scanned using a 20× magnification on the Aperio Digital Pathology System (Leica Microsystems, Wetzlar, Germany). To quantify staining intensity of each sample, a scoring system was implemented to analyze the immunohistochemical staining. Each section was scored using a staining index^44^ composed of the intensity of the staining on a scale of 0–3 and the extent of the staining on a scale of 0–100%. The final score of each sample was determined by multiplying the extent score by the intensity score; therefore, the final staining index ranged from 0 (no staining) to 3 (strong and extensive staining). ZAK staining score ≥1.0 was considered as “positive” and was used as the cutoff score for all further statistical analysis. ER, PR, and HER2 data by IHC were obtained with the commercial TMA slides.

### Statistical analysis

For comparisons, the two-tailed Student’s *t*-test, one-way analysis of variance with Tukey’s post hoc test and two-way analysis of variance with Bonferroni post-tests were performed as appropriate. Correlations between ZAK expression status and clinicopathological parameters were analyzed using the Chi-square test or Fisher’s exact test. The survival probability was evaluated using the Kaplan–Meier method, and differences were assessed using the log-rank test. ROC analysis was used to determine the prognostic value of the parameters. All above analyses were performed with GraphPad Prism 5.0 (GraphPad Softioare Inc., San Diego, CA, USA). The Cox proportional-hazards model (SPSS 22.0; Chicago, IL, USA) was used to perform univariate and multivariate (backward stepwise regression) analyses to determine relative risk and independent significance.

## Electronic supplementary material

Revision- Supplementary Methods Legends and References

Supplementary Figures-revision-2-submitted

Supplementary Table S1

Supplementary Table S2

Supplementary Table S3-S5
