# Supplementary material for: Mixed lineage kinase ZAK promotes epithelial–mesenchymal transition in cancer progression
Source: Cell Death Dis. 2018 Feb 2;9(2):143. doi: 10.1038/s41419-017-0161-x (PMC5833348; doi:10.1038/s41419-017-0161-x)
Supplement: Supplementary file 1 — Revision- Supplementary Methods Legends and References [file 41419_2017_161_MOESM1_ESM.docx]

**Mixed lineage kinase ZAK promotes epithelial-mesenchymal transition in cancer progression.** Linna Li *et al.*

**Supplementary Materials and Methods**

**Cell culture and reagents**

The immortalized human mammary gland epithelial cells HMLE was generously provided by Dr. Robert Weinberg.[^1^](#_ENREF_1) HMLE cells were cultured in DMEM/F12 (1:1) medium (Mediatech, Moorseville, NC USA) supplemented with 10 µg/ml insulin, 10 ng/ml hEGF, 0.5 µg/ml hydrocortisone, and 1% penicillin-streptomycin. PC3 prostate cancer cells, matching to the PC3 cells from ATCC (Manassas, VA, USA) by finger printing, were kindly provided by Dr. Isaiah Fidler.[^2^](#_ENREF_2) PC3 and SU86.86 pancreatic cancer cells (ATCC) were cultured in RPMI 1640 media (Mediatech), supplemented with 10% FBS (Gibco, Grand Island, NY, USA) and 1% penicillin-streptomycin (Mediatech). HEK-293T cells, as well as three high metastatic potential cancer cell lines MDA-MB-231 (breast cancer cell line obtained from ATCC), HCCLM3 (hepatocellular carcinoma cell line[^3^](#_ENREF_3) obtained from China Infrastructure of Cell Line Resources, Beijing, China) and CLY (colorectal cancer cell line[^4^](#_ENREF_4) established by our lab) were cultured in DMEM (Invitrogen, Irvine, CA, USA) with 10% FBS and 1% penicillin-streptomycin. The control siRNA, siRNAs against c-FOS (a gift from Drs. Qin Li and Jianming Xu) and siRNA against FOSL1 were originally purchased from Dharmacon ([Lafayette, CO](https://www.google.com/search?safe=active&rlz=1C1HLDY_enUS732US732&q=Lafayette+Colorado&stick=H4sIAAAAAAAAAOPgE-LSz9U3MM41SzI0UuIAsYssDS21tLKTrfTzi9IT8zKrEksy8_NQOFYZqYkphaWJRSWpRcUAAwbVmkQAAAA&sa=X&ved=0ahUKEwjI5qGh2PrWAhWJj1QKHas-DI8QmxMIhwEoATAS), USA) and transfected into PC3-ZAK, HMLE-ZAK and 293T-ZAK cells using Lipofectamine RNAiMAX (Invitrogen, Irvine, CA, USA), according to the manufacturers’ instructions. The MAPK pathway inhibitors U0126 and RO5126766 were purchased from Selleck (Houston, TX, USA) and used to treat ZAK-overexpressing cells as indicated.

**Western blotting analysis**

Cells were lysed in RIPA buffer supplemented with Complete Mini protease inhibitor cocktail and PhosSTOP phosphatase inhibitor cocktail (Roche Applied Science, Indianapolis, IN, USA). Protein concentrations were determined using Pierce BCA protein assay kit (Thermo Scientific, Pittsburgh, PA, USA). The samples were then separated by SDS-PAGE and transferred to PVDF membrane (Bio-Rad, Hercules, CA, USA). The membrane was blocked with 5% skim milk in TBST for 1h at room temperature, followed by incubation of a primary antibody overnight at 4°C. Primary antibodies used were as follows: anti-vimentin V9 (Abcam, Cambridge, UK); anti-E-cadherin, anti-Fibronectin and anti-N-cadherin (BD Pharmingen, San Diego, CA, USA); anti-ZEB1 H102, anti-CD44 IM7, anti-occludin, and anti-β-actin (Santa Cruz Biotechnology, Santa Cruz, CA, USA); anti-ESRP1/2(Rockland Immunochemicals, Limerick, PA, USA); anti-ZAK (Sigma). After washes, the membrane was incubated with HRP-conjugated secondary antibodies (Cell Signaling Technology, Danvers, MA, USA) for 1 h at room temperature. The blots were then detected by Pierce ECL Western Blotting Substrate (Thermo Scientific) on Blue Basic Autoradiography Film.

**Immunofluorescence assay**

Cells grown on Lab-Tek II Chamber Slide (Thermo Scientific) were fixed with 4% paraformaldehyde/PBS for 10 min, and permeabilized with 0.2% Trition-100/PBS for 10 min before blocking with blocking buffer (PBS + 5% BSA + 0.1% Tween-20) for 1 h. Cells were then incubated with primary antibody (diluted 1:200) overnight at 4°C. Primary antibodies used were as follows: anti-E-cadherin (BD Pharmingen) and anti-vimentin V9 (Abcam). After washing with PBS, secondary antibodies coupled to Alexa-488 or -594 (Invitrogen, Irvine, CA, USA) were used in the dark at room temperature for 1 h. Cell nuclei were then visualized with DRAQ5 (Invitrogen). Slides were mounted with SlowFade Gold anti-fade reagent (Invitrogen). Images were captured with the Leica TCS SP5 Confocal Microscope and LAS AF software (Buffalo Grove, IL, USA).

**Reverse transcription and quantitative PCR analysis**

RNA was isolated from cells using TRIzol reagent (Invitrogen). cDNA was generated by reverse transcription with iScript cDNA Synthesis Kit (Bio-Rad). Real time PCR was performed using SsoFast EvaGreen Supermix (Bio-Rad) in Bio-Rad CFX96 Real-Time PCR Detection System (Bio-Rad). For all RT-PCR analysis, either GAPDH or β-actin mRNA was used to normalize RNA input and expression levels were calculated according to the comparative CT method (ΔΔCT). Primers sequence used to amplify genes were as follows:

| ZAK-F | | 5’-ACTTTGGTGCCTCTCGGT-3’ | |
| --- | --- | --- | --- |
| ZAK-R | | 5’-TTCTGGGGCAACTGCTT-3’ | |
| CDH1-F | | 5’-GGCCAGGAAATCACATCCTA-3’ | |
| CDH1-R | | 5’-GGCAGTGTCTCTCCAAATCC-3’ | |
| VIM-F | | 5’-AAAGTGTGGCTGCCAAGAAC-3’ | |
| VIM-R | | 5’-AGCCTCAGAGAGGTCAGCAA-3’ | |
| ZEB1-F | | 5’-TGTTACCAGGGAGGAGCAGT-3’ | |
| ZEB1-R | | 5’-GCTTCATCTGCCTGAGCTTC-3’ | |
| CD44s-F | | 5’-AGGAGCAGCACTTCAGGAGGTTAC-3’ | |
| CD44s-R | | 5’-ACTGGGGTGGAATGTGTCTTGGTC-3’ | |
| CD44v8-9 -F | | 5’-caggtttggtggaagatttgg-3’ | |
| CD44v8-9 -R | | 5’-tgtcagagtagaagttgttggatgg-3’ | |
| ESRP1-F | | 5’-accaagccctccgacagt-3’ | |
| ESRP1-R | | 5’-tgcaggatttgcctgacat-3’ | |
| FOSL1-71F | 5'-GTCGAAGGCCTTGTGAACAG-3' | |  |
| FOSL1-71R | 5'-CTCCGGTTCCTGCACTTG-3' | |  |
| FOSL1-62F | 5'-ATCCACCCAACCCTATCTCC-3' | |  |
| FOSL1-62R | 5'-CAGAATGGCCTGGTCCAA-3' | |  |
| c-FOS-F | 5'-CTACCACTCACCCGCAGACT-3' | |  |
| c-FOS-R | 5'-AGGTCCGTGCAGAAGTCCT-3' | |  |

**FACS Analysis**

Cells were trypsinized, washed with ice-cold FACS buffer (PBS containing 2% FBS, 1% Pen/Strep, and 5 mM EDTA pH8.0), and then incubated with APC-conjugated anti-CD44 (clone G44-26) and PE-conjugated anti-CD24 (clone ML5) (BD Pharmingen) in the dark at 4°C for 30 min. After washing, cells were resuspended in FACS buffer containing 75 ng/ml DAPI and analyzed on a BD FACSAria II Flow Cytometer (BD Bioscience).

**Mammoshpere assay**

HMLE cells carrying empty vector or expressing ZAK were seeded at 5 × 10**^3^** cells per well of a 24-well Ultra Low Plate (Corning) and grown for 14 days in DMEM/F12 (1:1) medium (Mediatech) supplemented with 20 ng/ml EGF, 20 ng/ml FGF (Cell Signaling Technology), 4 µg/ml heparin (Sigma), 2% Serum-Free Supplement B27 (Invitrogen) and 1% methylcellulose. Wells were fed every 3 days with 0.5 ml media for 2 weeks. Then the mammospheres with diameter >75 μm were counted at indicated time.

**Multilineage differentiation assay**

For osteoblast differentiation, 1 × 10^4^ cells were cultured in NH OsteoDiff Medium (MiltenyiBiotec, Auburn, CA, USA) in 12-well plates for 35 days; and for adipocyte differentiation, 1 × 10^5^ cells were cultured in NH AdipoDiff Medium (MiltenyiBiotec) for 42 days. The medium was replaced every 3 days. After culturing, committed osteogenic cells and adipogenic cells were detected as previously described.[^5^](#_ENREF_5)^,^ [^6^](#_ENREF_6) Briefly, to test osteoblastic differentiation, cells were incubated with FAST BCIP/NBT (Sigma-Aldrich) to determine alkaline phosphatase (AP) activity, or analyzed by alizarin red S (Sigma-Aldrich) staining and silver nitrate (Von-Kossa) staining to determine calcium deposition and mineral deposition. As to adipocyte differentiation, oil red O dye (Sigma-Aldrich) staining was used to detect oil droplets formation.

**Promoter reporter luciferase assay**

Cells were transiently co-transfected with firefly luciferase reporter vectors and pJP1563-ZAK or vector control using TransIT-LT1 Transfection Reagent (MirusBio, Madison, WI, USA). As internal control for transfection efficiency, renilla luciferase construct was cotransfected with reporter vectors. After 40 h, luciferase activity was assessed with Dual-Luciferase Reporter Assay System (Promega, Madison, WI, USA).

**Supplementary Figure Legends**

**Supplementary Figure S1.** ZAK induces EMT in SU86.86 cells. (a) Changes in the expression of EMT markers by ZAK overexpression in SU86.86 cells. Top, Western blot analysis (top) and RT-PCR analysis (bottom) of EMT markers expression. Shown on Y-axis is log10 fold change of mRNA level induced by ZAK vs vector control. Error bars denote S.D. from triplicate. (b) ZAK induced migration in SU86.86 cells determined by Boyden chamber assay, showing representative photos (top) and quantification (bottom) of migration. Data are represented as mean ± S.D. of triplicate experiments. ****P* < 0.001 (Student’s *t*-test). (c) Proliferation curves of SU86.86 cells expressing ZAK or empty vector (EV) grown in regular medium or nutrition reduced medium. The number of viable cells was measured by AlamarBlue assay at different time points and data are expressed as viable cell number as fold changes from that at Day 1. Mean values ± S.D. from three independent experiments are shown. **P* < 0.05 and ****P* < 0.001 (Two-way ANOVA followed by Bonferroni post-tests). (d) Dose-response curves of the above cell lines treated with chemotherapeutic drug. IC50 values were obtained by using logistic nonlinear regression analyzing model of MicroCal Origin 8.5 software. Error bars denote SD from two independent experiments, each done in quadruplicate. ***P* < 0.01 (Student’s *t*-test).

**Supplementary Figure S2.** **Candidate downstream signaling molecules underlying ZAK-induced EMT.** (a) Silencing of AP1 genes (c-FOS and FOSL1) in ZAK-overexpressing cells did not show concordant expression changes of EMT markers. Two doses of siRNA were used (2ul and 4ul) per well in 6-well plates. (b) Scatter plots obtained from cBioPortal TCGA database in August, 2016 show positive correlation between ZAK and ZEB1 mRNA expression. Pearson’s correlation coefficients and *P* values are indicated. The TCGA Provisional datasets for each indicated cancer types were accessed in August 2016 and used for the analyses. (c) Western blot analysis for ZEB1 and ZAK protein levels upon ZAK overexpression in HMLE cells. (d) Western blot analysis of ZEB1 and its target CDH1 upon expression of ZEB1 shRNA in PC3-ZAK and 293T-ZAK cells. (e) Western blot analysis of EMT proteins in PC3-ZAK and 293T-ZAK cells treated with either DMSO control, or MAPK pathway inhibitors, either U0126 (10uM) or RO5126766 (10uM) for 24 hr. (f) ZAK wild type cDNA can, while ZAK kinase dead cDNA (ZAK-K45M) can not, induce EMT gene expression changes.

**Supplementary Figure S3.** Silencing of ZAK gene in HCCLM3 and CLY cells results in reversal of EMT. (a) Western blot analysis of ZAK, E-cadherin, N-cadherin and ZEB1 in cancer cell lines. (b) Changes in the expression of ZAK and EMT-associated genes by ZAK knockdown as determined by real time PCR. Shown on Y-axis is log10 fold change of mRNA level induced by shZAKs vs scramble control. Error bars denote S.D. from triplicate. (c) ZAK depletion reduced migration of the two mesenchymal cell lines determined by Boyden chamber assay, showing representative photos (top) and quantification (bottom) of migration. Quantification of migration is shown as means (S.D. from three independent experiments). (d) Proliferation curves of the above cells grown in regular media (left) or nutrition reduced media (right). Mean values ± S.D. from three independent experiments are shown. **P* < 0.05, ***P* < 0.01, and ****P* < 0.001 (Two-way ANOVA followed by Bonferroni post-tests). (e) Dose-response curves of the above cells treated with graded concentrations of paclitaxel. IC50 values were obtained by using logistic nonlinear regression analyzing model of MicroCal Origin 8.5 software. Error bars denote S.D. from two independent experiments, each done in quadruplicate. **P* < 0.05 and ***P* < 0.01 (One-way ANOVA followed by Tukey’s test).

**Supplementary Figure S4.** shRNA silencing of ZAK gene in MDA-MB-231 cells has no influence on subcutaneous tumor formation in immunodeficient mice. Representative 2D bioluminescence images of nude mice subcutaneously transplanted with MDA-MB-231-shCtrl cells or MDA-MB-231-shZAK2 cells (n=6). Changes in tumor growth were calculated and expressed as fold changes of bioluminescence values at different time points relative to that of Day 0. Data are represented as mean ± S.D. No significant difference was found between the two groups, according to Two-way ANOVA followed by Bonferroni post-tests.

**Supplementary Figure S5.** Cross-cancer alteration summary for ZAK obtained from cBioPortal TCAA database ([www.cbioportal.org](http://www.cbioportal.org)) in August, 2016.

**Supplementary Figure S6.** ZAK overexpression is significantly associated with poor prognosis (a) Comparison of ZAK mRNA expression between altered group and unaltered group in 3 TCGA datasets. (b) Kaplan-Meier survival curves depending on ZAK mRNA alterations in above TCGA datasets. All plots and statistical analyses in (a) and (b) were obtained from cBioPortal TCGA database ([www.cbioportal.org](http://www.cbioportal.org)) in August, 2016.

**Supplementary Figure S7.**Combination of ZAK with clinicopathological parameters results in more accurate stratification and prognosis of breast invasive cancer cases within the TMA dataset. (a) Kaplan-Meier overall survival curves depending on individual or combined clinicopathological parameters. (b) Kaplan-Meier overall survival curves depending on the combination of ZAK with clinicopathological parameters. The *P*-value was calculated using the Log-rank test. AUCs obtained from ROC analysis represented the prognostic accuracy.

**Supplementary Reference**

1. Elenbaas B, Spirio L, Koerner F, Fleming MD, Zimonjic DB, Donaher JL *et al.* Human breast cancer cells generated by oncogenic transformation of primary mammary epithelial cells. *Genes Dev* 2001; **15:** 50-65.

2. Pettaway CA, Pathak S, Greene G, Ramirez E, Wilson MR, Killion JJ *et al.* Selection of highly metastatic variants of different human prostatic carcinomas using orthotopic implantation in nude mice. *Clin Cancer Res* 1996; **2:** 1627-1636.

3. Li Y, Tang Y, Ye L, Liu B, Liu K, Chen J *et al.* Establishment of a hepatocellular carcinoma cell line with unique metastatic characteristics through in vivo selection and screening for metastasis-related genes through cDNA microarray. *J Cancer Res Clin Oncol* 2003; **129:** 43-51.

4. Li LN, Zhang HD, Yuan SJ, Tian ZY, Sun ZX. Establishment and characterization of a novel human colorectal cancer cell line (CLY) metastasizing spontaneously to the liver in nude mice. *Oncol Rep* 2007 **17:** 835-840.

5. Battula VL, Evans KW, Hollier BG, Shi Y, Marini FC, Ayyanan A *et al.* Epithelial-mesenchymal transition-derived cells exhibit multilineage differentiation potential similar to mesenchymal stem cells. *Stem Cells* 2010; **28:** 1435-1445.

6. Koch TG, Heerkens T, Thomsen PD, Betts DH. Isolation of mesenchymal stem cells from equine umbilical cord blood. *BMC Biotechnol* 2007; **7:** 1-9.
