# Supplementary material for: Mixed lineage kinase ZAK promotes epithelial–mesenchymal transition in cancer progression
Source: Cell Death Dis. 2018 Feb 2;9(2):143. doi: 10.1038/s41419-017-0161-x (PMC5833348; doi:10.1038/s41419-017-0161-x)
Supplement: Supplementary file 2 — Supplementary Figures-revision-2-submitted [file 41419_2017_161_MOESM2_ESM.pptx]

## Slide 1
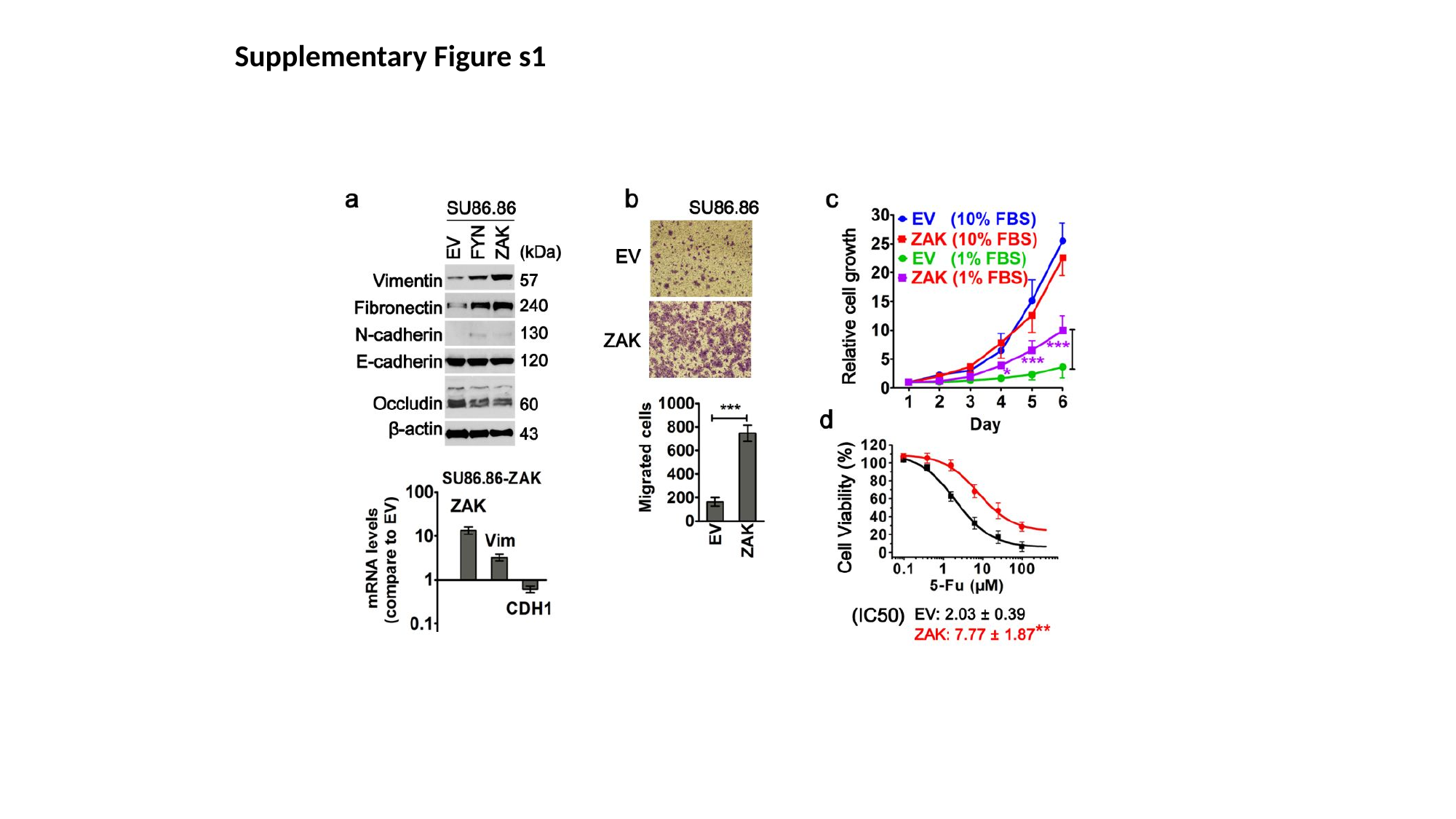

Supplementary Figure s1

## Slide 2
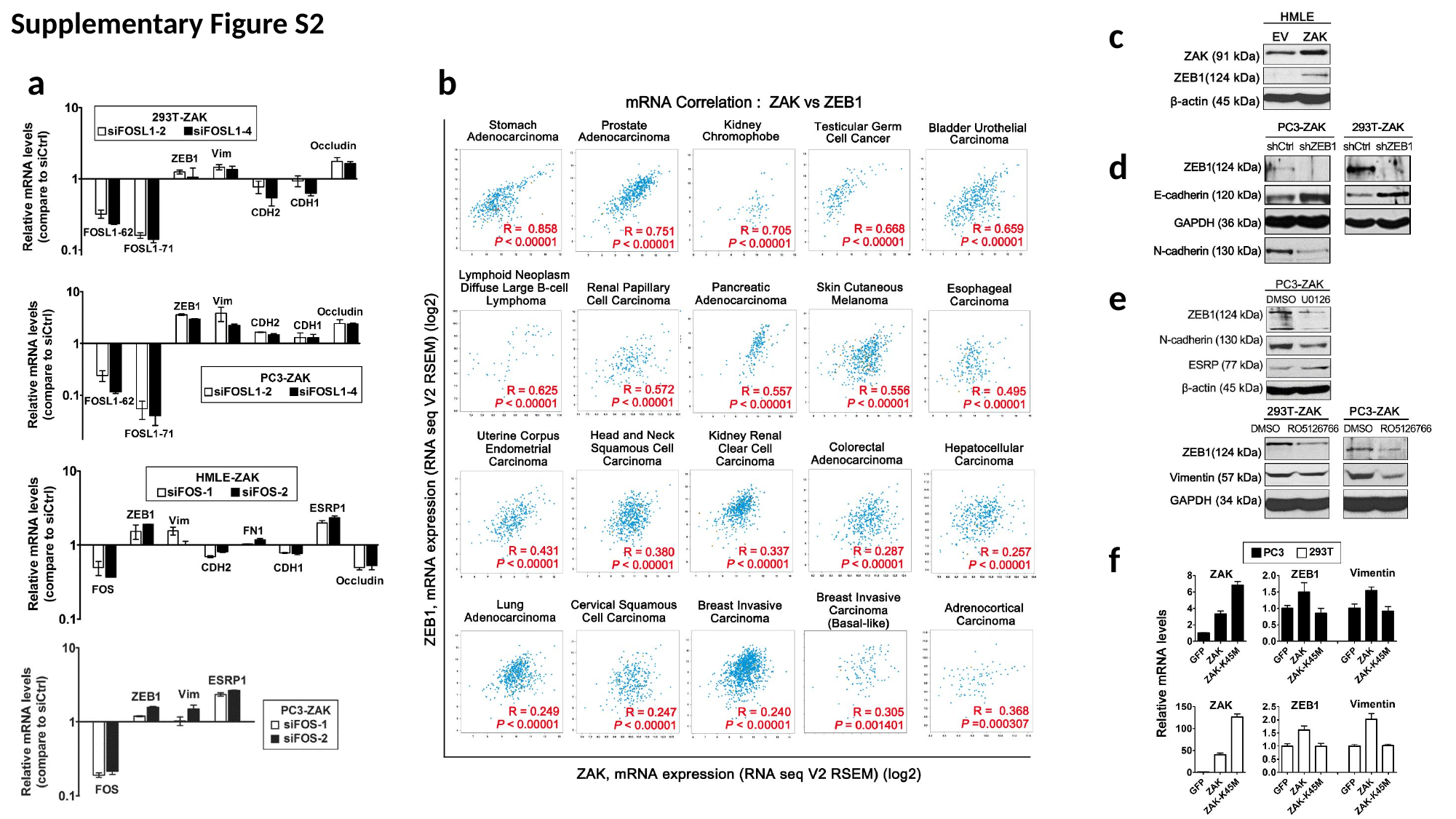

Supplementary Figure S2
c
d
e
f
b
a

## Slide 3
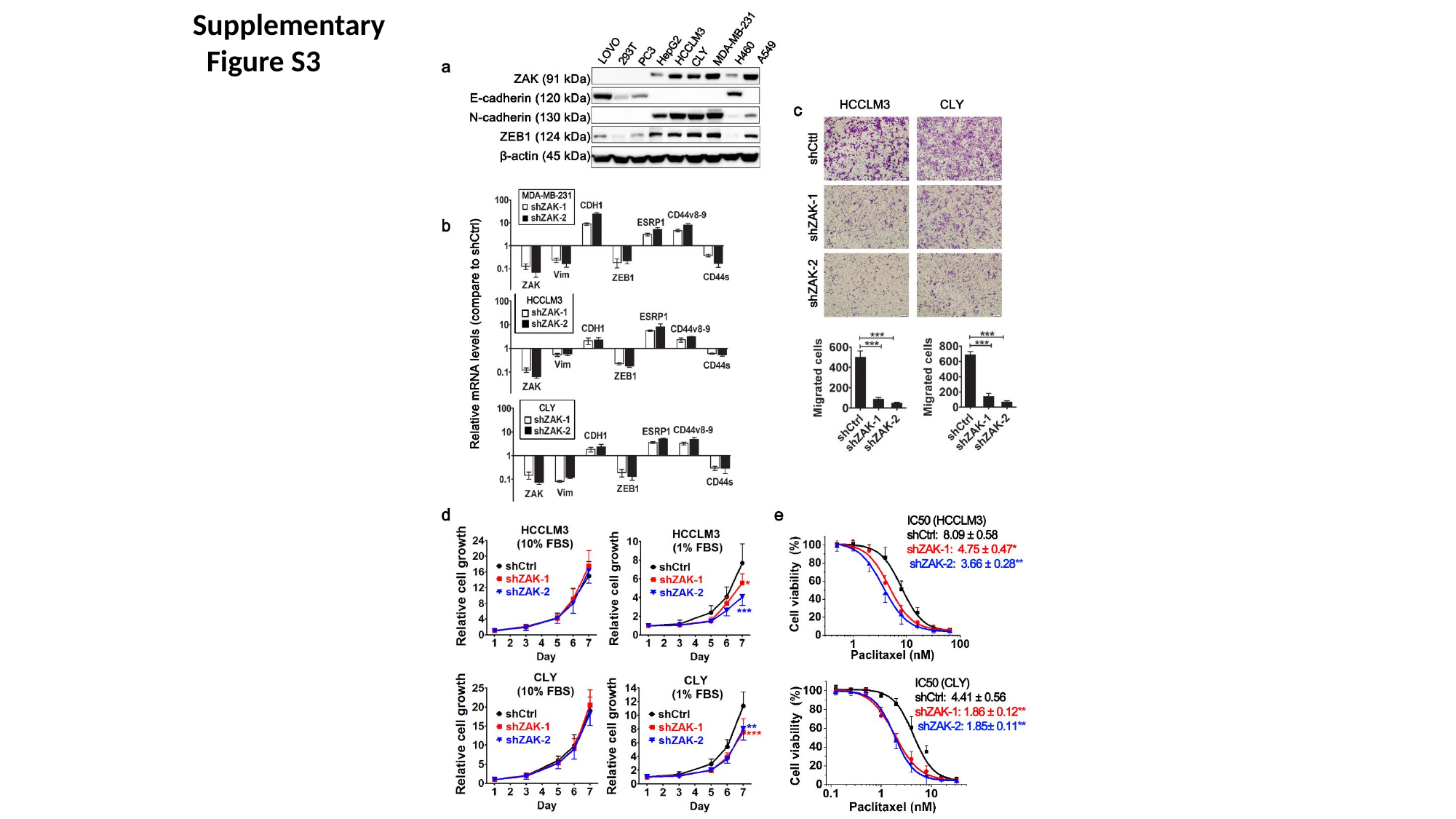

Supplementary
 Figure S3

## Slide 4
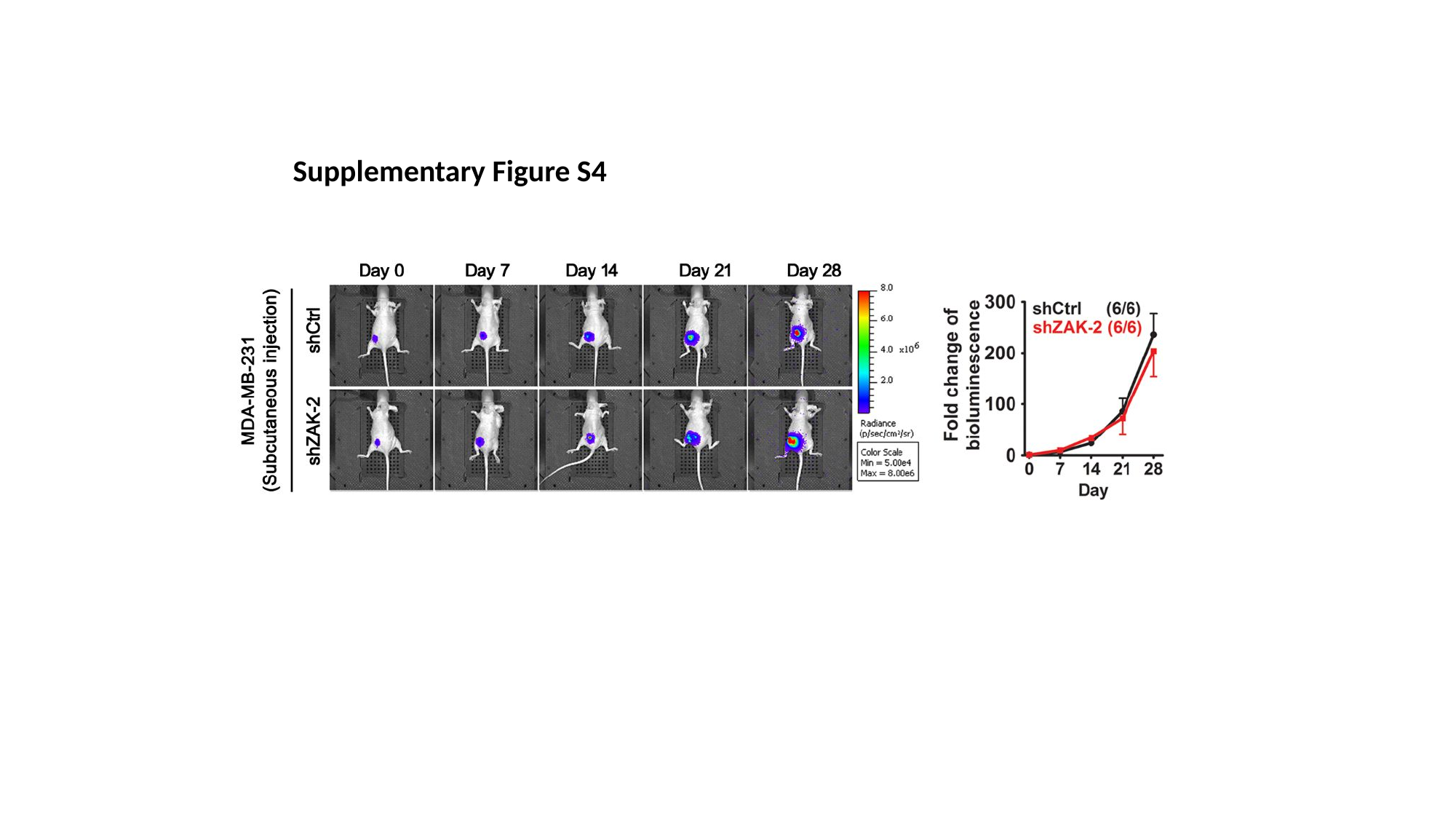

Supplementary Figure S4

## Slide 5
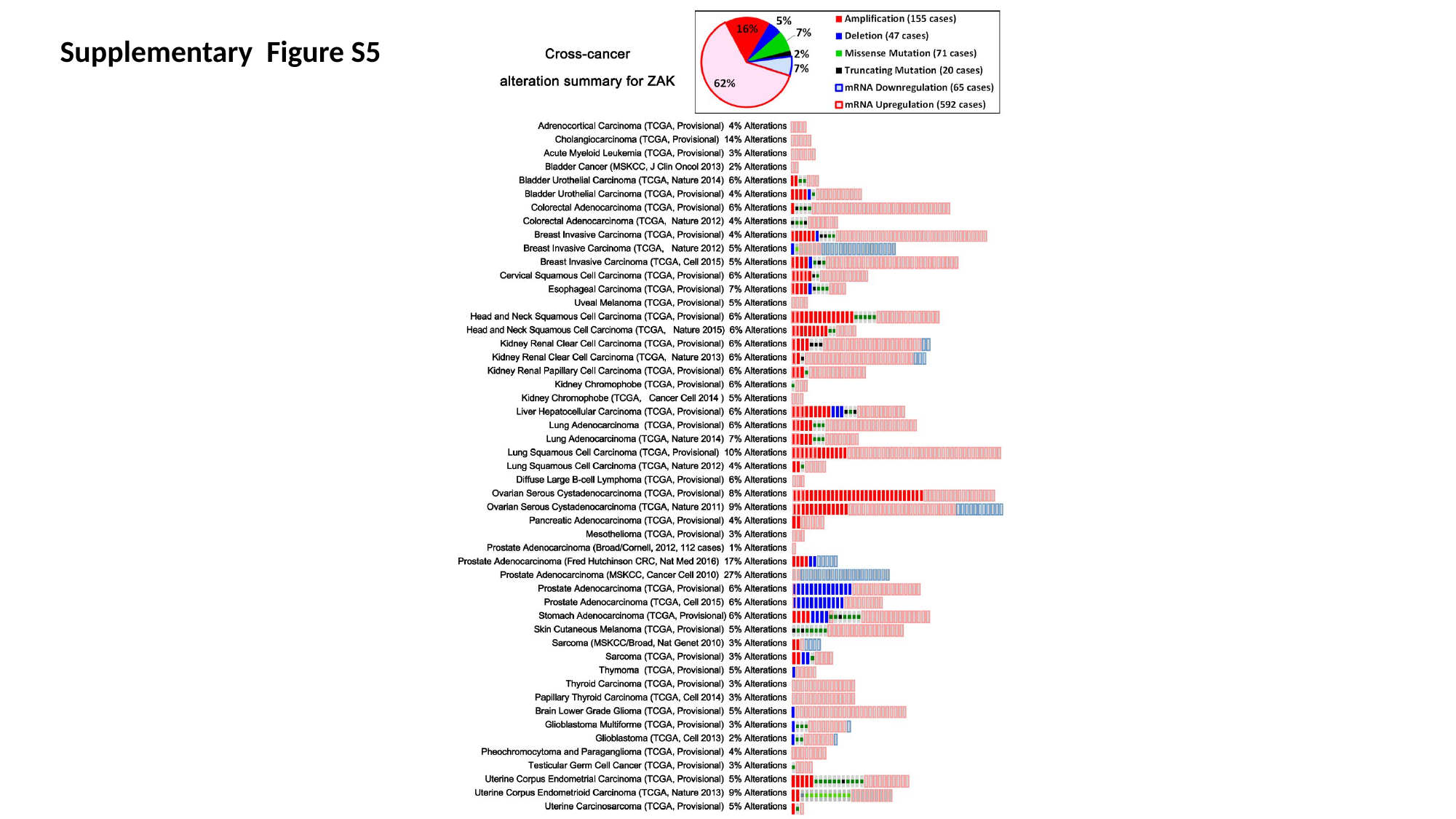

Supplementary Figure S5

## Slide 6
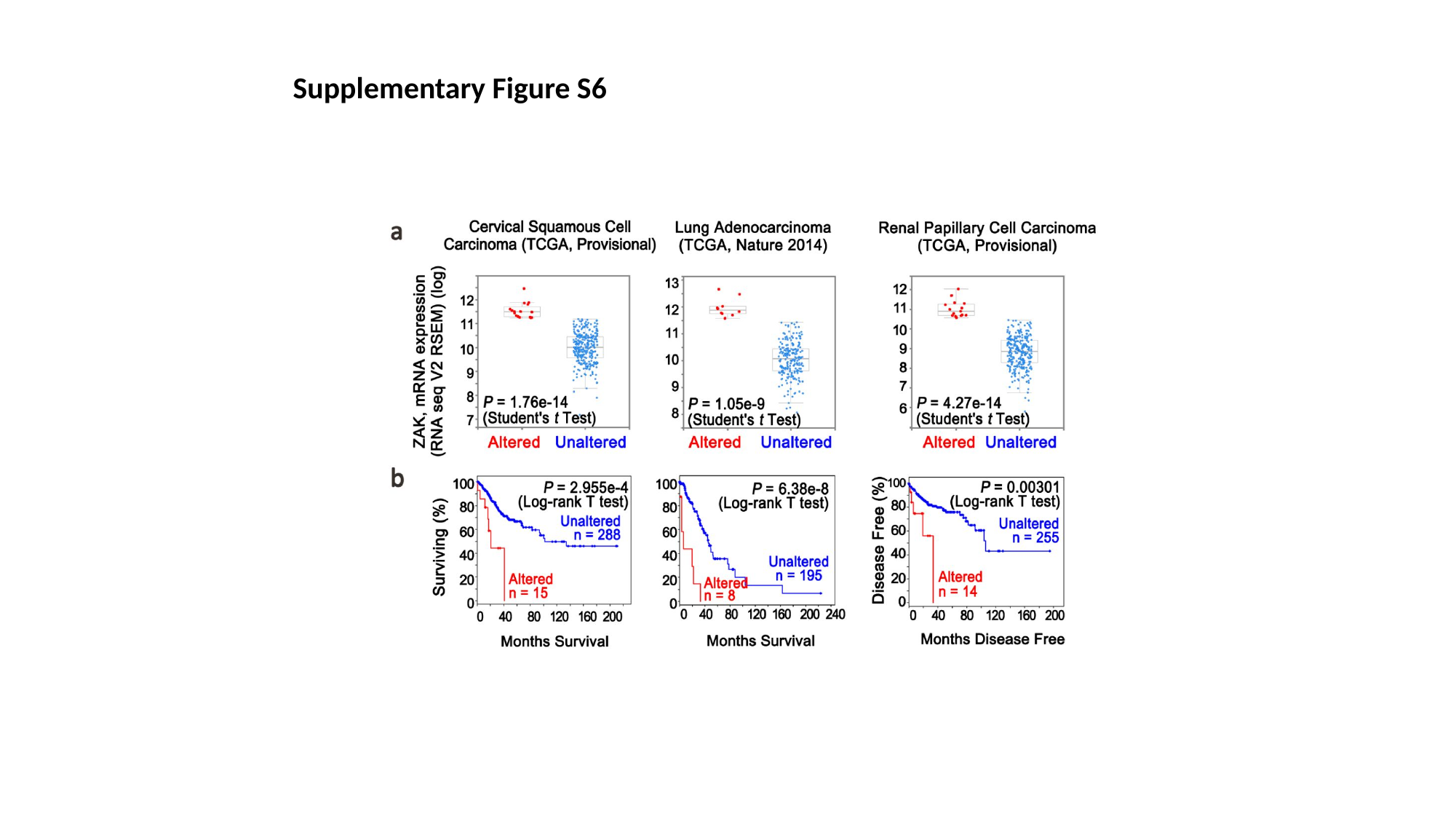

Supplementary Figure S6

## Slide 7
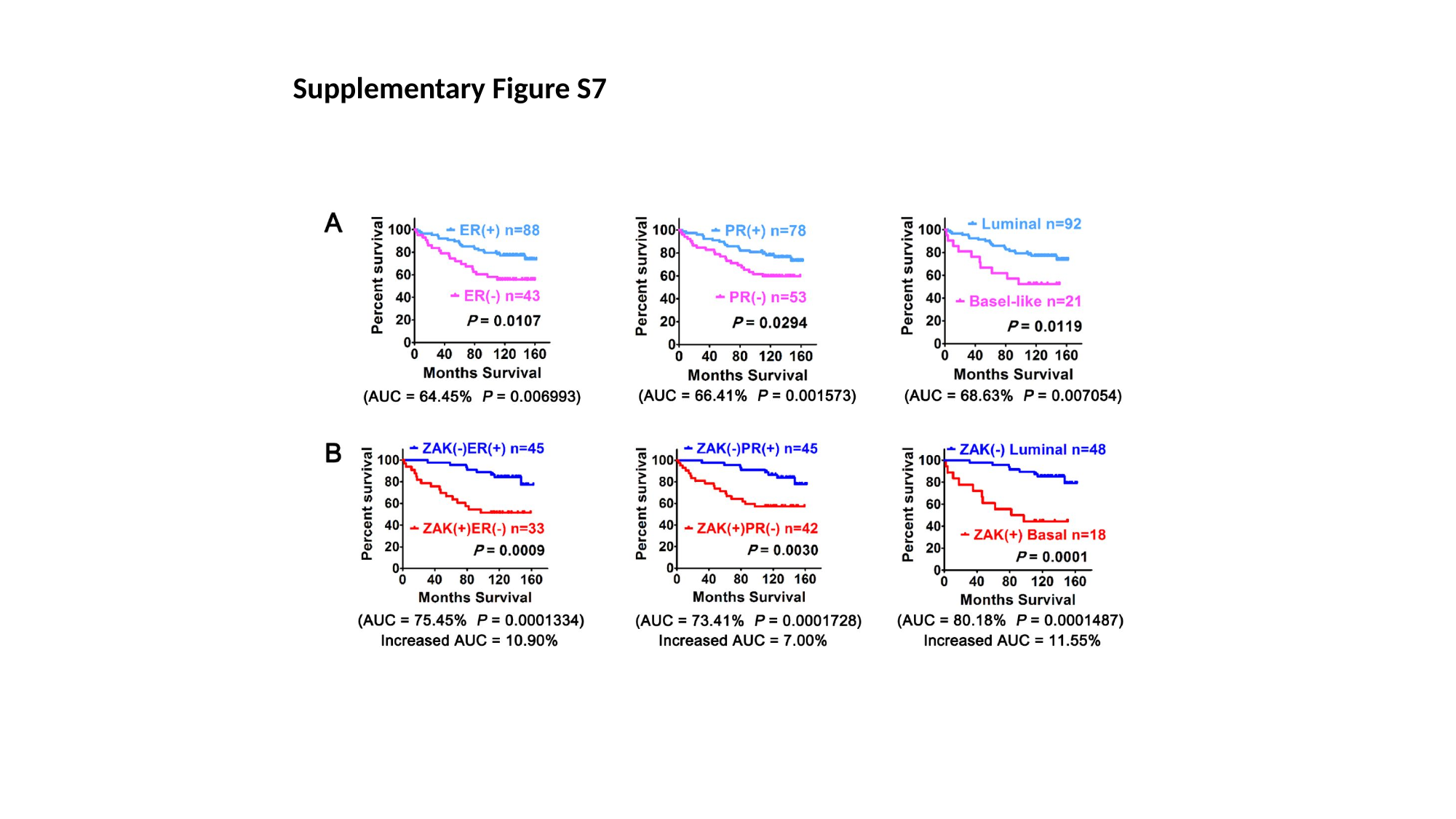

Supplementary Figure S7
