# Supplementary material for: Mixed lineage kinase ZAK promotes epithelial–mesenchymal transition in cancer progression
Source: Cell Death Dis. 2018 Feb 2;9(2):143. doi: 10.1038/s41419-017-0161-x (PMC5833348; doi:10.1038/s41419-017-0161-x)
Supplement: Supplementary file 5 — Supplementary Table S3-S5 [file 41419_2017_161_MOESM5_ESM.pdf]

Table S3 Basic demographic data for 140 evaluable breast cancer cases

**Clinical characteristics**	Number (n)	Percent (%)
**AJCC tumor classification**		
Stage I	11	8.0
Stage II	80	58.0
Stage III	47	34.1
**Primary tumor stage**		
pT1	30	21.4
pT2	96	68.6
pT3	14	10.0
**Lymph node status**		
pN0	52	37.7
pN1	42	30.4
pN2	35	25.4
pN3	9	6.5
**Intrinsic subtype**		
Luminal A and B (ER+ and/or PR+)	92	71.3
HER2 type (ER-,PR-,HER2+)	16	12.4
Basal-like (ER-,PR-,HER2-)	21	16.3

Table S4 Association between ZAK expression and clinicopathological parameters

Clinicopathological parameters	ZAK (-) n (%)	ZAK (+) n (%)	*P* value [a]	Relative risk [b] (95% CI)
**AJCC classification**			0.7130	1.091 (0.7031-1.692)
Stage I-II	35 (27.3)	47 (36.7)		
Stage III	18 (14.1)	28 (21.9)		
**Primary tumor stage**			0.3922	1.539 (0.6554-3.613)
pT1-2	51 (39.5)	65 (50.8)		
pT3	4 (3.1)	10 (7.8)		
**Lymph node status**			0.850	1.071 (0.6863-1.672)
pN0-1	36 (28.1)	49 (38.3)		
pN2-3	17 (13.3)	26 (20.3)		
**Estrogen-receptor**			**0.0002**	**0.3556 (0.1852-0.6825)**
ER-	8 (6.5)	33 (26.8)		
ER+	45 (36.6)	37 (30.1)		
**Progesterone-receptor**			**<0.0001**	**0.2596 (0.1341-0.5025)**
PR-	8 (6.5)	42 (34.1)		
PR+	45 (36.6)	28 (22.8)		
**HER2**			**0.0214**	**1.793 (1.063-3.024)**
HER2-	42 (33.9)	40 (32.3)		
HER2+	12 (9.7)	30 (24.2)		
**Intrinsic subtype**			**<0.0001**	**0.2488 (0.1080-0.5736)**
HER2 type (ER-PR-HER2+) & Basal-like (ER-PR-HER2-)	5 (4.1)	31 (25.4)		
Luminal A and B (ER+ and/or PR+)	48 (39.3)	38 (31.1)		

[a] Fisher's exact test

[b] Relative risk represents the strength of association between ZAK and other biomarkers. Relative risk = 1 (no correlation); Relative risk > 1 (positive correlation); Relative risk < 1 (negative correlation).

95% CI: 95% confidence intervals.

Table S5 COX univariate and multivariate analysis for the effect of clinicopathologic parameters and ZAK expression on overall survival

Clinicopathological parameters	Univariate analyses		Multivariate analyses	
	**Hazard ratio (95% CI)**	***P* value**	**Hazard ratio (95% CI)**	***P* value**
**AJCC classification 1**				
Stage I [a]	1		1	
Stage II	3.167 (0.426-23.552)	0.260	3.428 (0.457-25.742)	0.231
Stage III	6.867 (0.925-50.963)	0.060	7.454 (0.994-55.888)	0.051
**AJCC classification 2**				
Stage I-II [a]	1		1	
Stage III	**2.381 (1.318-4.302)**	**0.004****	**2.447 (1.293-4.631)**	**0.006****
**Primary tumor stage 1**				
pT1 [a]	1		1	
pT2	1.447 (0.637-3.286)	0.377	1.132 (0.470-2.727)	0.783
pT3	2.471 (0.829-7.366)	0.104	1.292 (0.382-4.363)	0.680
**Primary tumor stage 2**				
pT1-2 [a]	1		1	
pT3	1.849 (0.780-4.384)	0.163	1.163 (0.436-3.102)	0.763
**Lymph node status 1**				
pN0 [a]	1		1	
pN1	1.000 (0.438-2.282)	0.999	1.155 (0.478-2.791)	0.748
pN2	**2.441 (1.185-5.027)**	**0.016***	**2.770 (1.269-6.048)**	**0.011***
pN3	2.202 (0.781-6.758)	0.168	2.051 (0.571-7.364)	0.271
**Lymph node status 2**				
pN0-1 [a]	1		1	
pN 2-3	**2.391 (1.323-4.322)**	**0.004****	**2.458 (1.298-4.656)**	**0.006****
**Biomarkers**				
ZAK (+)	**2.726 (1.368-5.435)**	**0.004****	**2.775 (1.344-5.730)**	**0.006****

ER (+)	**0.455 (0.244-0.846)**	**0.013***	0.843 (0.313-2.275)	0.737
PR (+)	**0.507 (0.272-0.946)**	**0.033***	0.647 (0.317-1.324)	0.233
HER2 (+)	1.023 (0.529-1.977)	0.947	0.648 (0.322-1.305)	0.224
**Combination 1**				
ZAK- ER+ [a]	1		1	
ZAK- ER-	1.369 (0.290-6.454)	0.691	1.463 (0.308-6.956)	0.632
ZAK+ ER+	2.202 (0.990-5.392)	0.084	2.339 (0.947-5.779)	0.066
ZAK+ ER-	**3.779 (1.614-8.849)**	**0.002****	**3.417 (1.458-8.010)**	**0.005****
**Combination 2**				
ZAK- PR+ [a]	1		1	
ZAK- PR-	2.277 (0.603-8.597)	0.225	1.542 (0.319-7.460)	0.243
ZAK+ PR+	2.512 (0.990-6.372)	0.053	**2.659 (1.007-7.020)**	**0.048***
ZAK+ PR-	**3.288 (1.426-7.585)**	**0.005****	**3.654 (1.514-8.821)**	**0.004****
**Combination 3**				
ZAK- HER2- [a]	1		1	
ZAK- HER2+	1.396 (0.370-5.268)	0.623	1.337 (0.342-5.227)	0.677
ZAK+ HER2+	1.997 (0.769-5.187)	0.156	2.050 (0.762-5.514)	0.155
ZAK+ HER2-	**3.401 (1.485-7.786)**	**0.004****	**3.637 (1.518-8.717)**	**0.004****
**Combination 4**				
ZAK- Luminal type [a]	1		1	
ZAK+ Luminal type	2.164 (0.924-5.069)	0.076	2.305 (0.976-5.446)	0.057
ZAK+ HER2 type	2.682 (0.897-8.012)	0.077	**2.324 (0.773-6.985)**	**0.133**
ZAK+ Basal-like	**4.509 (1.825-11.144)**	**0.001****	**4.357 (1.759-10.791)**	**0.001****
**Combination 5**				
ZAK- pN0-1 [a]	1		1	
ZAK- pN2-3	1.761 (0.537-5.772)	0.350	1.567 (0.442-5.558)	0.487
ZAK+ pN0-1	2.130 (0.826-5.497)	0.118	2.021 (0.776-5.266)	0.150
ZAK+ pN2-3	**6.538 (2.548-16.774)**	**<0.001*****	**5.831 (2.234-15.224)**	**<0.001*****

**Combination 6**				
ZAK- pT1-2 [a]	1		1	
ZAK- pT3	0.000 (0.000-4.267)	0.974	0.000 (0.000-6.395)	0.974
ZAK+pT1-2	**2.224 (1.093-4.572)**	**0.028***	**2.129 (1.007-4.501)**	**0.048***
ZAK+pT3	**5.313 (1.958-14.418)**	**0.001****	**5.482 (1.984-15.147)**	**<0.001*****
**Combination 7**				
ZAK- AJCC I-II [a]			1	
ZAK- AJCC III	1.596 (0.487-5.231)	0.440	1.408 (0.397-4.990)	0.596
ZAK+ AJCC I-II	1.980 (0.760-5.157)	0.162	1.871 (0.710-4.926)	0.205
ZAK+ AJCC III	**6.221 (2.444-15.835)**	**<0.001*****	**5.574 (2.156-14.411)**	**<0.001*****

[a] reference group

Luminal type: ER+ and/or PR+; HER2 type: ER-PR-HER2+; Basal like: ER-PR-HER2-.

95% CI: 95% confidence intervals
